# Adipocyte enhancer binding protein 1 knockdown alleviates osteoarthritis through inhibiting NF‐κB signaling pathway‐mediated inflammation and extracellular matrix degradation

**DOI:** 10.1002/ccs3.12022

**Published:** 2024-03-22

**Authors:** Le Cao, Weilu Gao, Haitao Yang, Ran Zeng, Zongsheng Yin

**Affiliations:** ^1^ Department of Orthopedics The First Affiliated Hospital of Anhui Medical University Hefei Anhui China; ^2^ Department of Orthopedics Fuyang Hospital of Anhui Medical University Fuyang Anhui China; ^3^ Department of Intensive Care Unit Fuyang Hospital of Anhui Medical University Fuyang Anhui China

**Keywords:** adipocyte enhancer binding protein 1, extracellular matrix, inflammation, osteoarthritis

## Abstract

Inflammation promotes the degradation of the extracellular matrix, which contributes to the development of osteoarthritis (OA). Adipocyte enhancer binding protein 1 (AEBP1) participates in multiple pathological processes related to inflammatory diseases. However, the role of AEBP1 in OA development is unknown. We found a higher AEBP1 expression in articular cartilage of OA patients (*n* = 20) compared to their normal controls (*n* = 10). Thus, we inferred that AEBP1 might affect OA progression. Then mice with destabilization of the medial meniscus (DMM) surgery and chondrocytes with IL‐1β treatment (10 ng/mL) were used to mimic OA. The increased AEBP1 expression was observed in models of OA. AEBP1 knockdown in chondrocytes reversed IL‐1β‐induced inflammation and extracellular matrix degradation, which was mediated by the inactivation of NF‐κB signaling pathway and the increased IκBα activity. Co‐immunoprecipitation assay indicated the interaction between AEBP1 and IκBα. Importantly, IκBα knockdown depleted the protective role of AEBP1 knockdown in OA. Moreover, AEBP1 knockdown in mice with OA showed similar results to those in chondrocytes. Collectively, our findings suggest that AEBP1 knockdown alleviates the development of OA, providing a novel strategy for OA treatment.

## Graphical abstract

Adipocyte enhancer binding protein 1 was highly expressed in osteoarthritis (OA) samples and its knockdown protected against inflammation and extracellular matrix degradation in the pathological state of OA by suppressing the activation of NF‐κB signaling pathway.

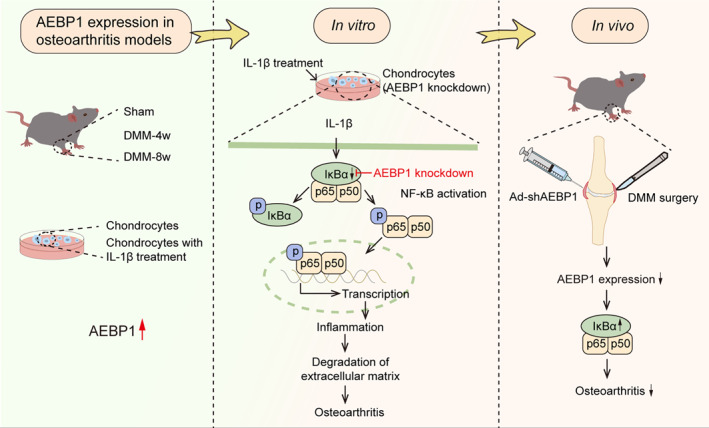

## 1 INTRODUCTION

Osteoarthritis (OA) is an articular cartilage disease affecting the entire joint structure and characterized by the loss of articular cartilage and other joint disruption, leading to the typical clinical symptoms of pain, stiffness, joint dysfunction, and loss of valued activities.^1^, ^2^ It is estimated that approximately 12% of people over 25 and 50% of people over 65 years of age were affected by OA.^3^ About 240 million people are involved in symptomatic, activity‐limiting OA worldwide.^1^, ^4^ OA has become the most prevalent arthritic disease and a leading cause of disability, and it has given rise to a heavy burden on society owing to the extensive costs for treatment and prevention, and incapacity to work.^5^ The pathogenesis of OA is related to multiple factors. Age, obesity, sex, joint injury, and inflammation are considered risk factors for OA.^4^, ^6^ As traditional palliative treatments cannot prevent further joint degeneration,^7^ it is imperative to explore novel therapies for alleviating the destruction and the global burden caused by OA.

Structural destruction and dysfunction of the articular cartilage belong to hallmark features of OA pathology.^8^ Articular cartilage is composed of a dense extracellular matrix and chondrocytes embedded in the extracellular matrix.^9^ It functions as a lubricating and load‐bearing surface in joints.^10^ Chondrocytes synthesize extracellular matrix proteins,^11^ including type II collagens, proteoglycans, other collagens, and non‐collagenous proteins,^12^ which are important for elasticity and stiffness.^13^ Articular cartilage homeostasis requires a balance between degradation and synthesis of extracellular matrix components. Disturbed homeostasis presents when the degradation of the extracellular matrix is faster than the synthetic capacity of chondrocytes, thereby promoting the development of OA.^14^ Thus, maintaining the function of chondrocytes and the balance of the extracellular matrix might be the key to regulating the development of OA.

Adipocyte enhancer binding protein 1 (AEBP1) was implicated in multiple pathological processes through regulating inflammation, one of the risk factors of OA. Ren et al.^15^ reported that AEBP1 was associated with the activated NF‐κB signaling pathway, which elevated the expression of pro‐inflammatory cytokines. AEBP1 overexpression also was proved to be able to activate NF‐κB signaling pathway.^16^ Moreover, AEBP1 could encode aortic carboxypeptidase‐like protein, which was associated with the extracellular matrix.^17^ All these previous studies indicated that AEBP1 was closely related to cellular inflammation and the formation of the extracellular matrix. Importantly, through database analysis, we found that a high expression level of AEBP1 was positively associated with the development of OA.

Therefore, we supposed that AEBP1 might play a role in OA progression. Then the in vivo and in vitro models of OA were constructed to explore the association between AEBP1 expression and OA progression.

## 2 MATERIALS AND METHODS

### 2.1 GSE dataset analysis

To preliminarily determine the AEBP1 expression in OA samples, we downloaded and analyzed three datasets (GSE169077, GSE8077, GSE53857) from the NIH‐GEO dataset database (http://www.ncbi.nlm.nih.gov/gds/). The GSE169077 dataset contained gene expression profiles of knee cartilage samples from normal participants and patients with OA. The GSE8077 dataset contained gene expression profiles of articular chondrocytes collected from the knees of OA‐ or sham‐operated rats. The GSE53857 dataset contained gene expression profiles of articular cartilage collected from the medial tibial plateau of untreated or OA‐operated mice at 2, 4, and 8 weeks post‐surgery.

### 2.2 Patients and sample collection

Patients with knee OA were recruited for collection of knee joints. Those patients with femoral neck fractures were used as controls. According to the Kellgren‐Lawrence scale system. Grade III or IV was the criterion for the OA group. Grade 0, I, or no obvious clinical features was the criterion for the normal group (the N group). No statistical difference was found in age between the two groups, demonstrating that patients with OA are age‐matched with their controls. All patients who participated in our study have signed informed consent. The experiment protocols were performed according to the Declaration of Helsinki. Information on human samples was listed in (Table S1).

### 2.3 Cell isolation and culture

Human chondrocytes were isolated according to previous studies.^18^, ^19^ Briefly, articular cartilage tissues were cut into 1 mm^3^ pieces and digested with 0.25% trypsin (Beyotime) for 30 min and 0.2% type II collagenase (Sigma) at 37°C overnight. Then human chondrocytes were resuspended and cultured in Dulbecco's modified eagle medium (DMEM)/F12 (Biosharp) containing 10% fetal bovine serum (FBS) and 1% penicillin‐streptomycin.

### 2.4 Adenovirus infection

The shRNA targeting AEBP1 or IκBα was inserted into the pShuttle‐CMV vector (Fenghui). Following PacI (Thermo Fisher) linearization, the shuttle vector was transformed into BJ5183‐AD‐1 chemically competent cells containing the adenoviral backbone plasmid (WeidiBio) to extract the recombinant adenoviral DNA, which was transfected into HEK293A cells (iCell Bioscience) using Lipofectamine 3000 (Invitrogen) to generate the recombinant adenovirus carrying shAEBP1 or shIκBα (Ad‐shAEBP1 or Ad‐shIκBα). HEK293A cells were cultured with DMEM (Servicebio) supplemented with 10% FBS in an incubator at 37°C with 5% CO_2_. For gene knockdown, chondrocytes were infected using the culture medium containing the recombinant adenovirus (MOI = 100) and cultured in an incubator at 37°C with 5% CO_2_. After infection, chondrocytes were collected at different time points for subsequent experiments. AEBP1 knockdown and IκBα knockdown in chondrocytes were performed through co‐infection of adenoviruses carrying shAEBP1 and adenoviruses carrying shIκBα (Ad‐shIκBα). After being co‐infected with adenoviruses, chondrocytes were collected for subsequent experiments. The sequence for AEBP1 shRNA was as follows: GGACTACAATGACCAGATAGA (GENERAL). The sequence for IκBα shRNA was as follows: GGACGAGGAGTACGAGCAAAT (GENERAL).

### 2.5 Cell treatment

Human chondrocytes were treated with human IL‐1β (10 ng/mL) for 0, 6, 12, or 24 h to detect the expression level of AEBP1. After infection, chondrocytes were treated with human IL‐1β (10 ng/mL) for 24 h to construct the OA model in vitro. After infection, chondrocytes were treated with cycloheximide (CHX) (10 ng/mL) (Aladdin) for 0, 1, 2, 3, or 4 h to determine the protein level of IκBα. After co‐infection, chondrocytes were treated with human IL‐1β for 24 h for subsequent experiments.

### 2.6 Animal experiments

Male C57BL/6J mice aged 10–12 weeks (Liaoning Changsheng) were randomly divided into three groups: the Sham group, the DMM‐4w group, and the DMM‐8w group. After being anaesthetized, mice were subjected to the destabilization of the medial meniscus (DMM) surgery to induce OA. Briefly, the longitudinal skin incision was made at the medial side of the knee joint of mice to expose the patellar tendon. Subsequently, the patellar tendon was dissected and the joint capsule immediately medial to patellar tendon was opened to expose the medial meniscotibial ligament (MMTL). Following that, a transection of the MMTL was performed to induce DMM. Mice without transecting the MMTLs were used as the Sham group. After DMM surgery, mice were subjected to euthanasia after continued feeding for 4 or 8 weeks. Knee joints of mice were taken for follow‐up experiments.

Male C57BL/6J mice aged 10–12 weeks (Liaoning Changsheng) were randomly divided into four groups: the Sham group, the DMM group, the DMM + Ad‐shNC group, and the DMM + Ad‐shAEBP1 group. After being anaesthetized, mice were subjected to DMM surgery to induce OA. Mice without transecting the MMTLs were used as the Sham group. The 10 μL (5 × 10^9^ PFU) of Ad‐shNC or Ad‐shAEBP1 were administered into mice through intra‐articular injection using a micro syringe (50 μL of Hamilton 705 RN SYR) at 10 days after DMM surgery. The injection was performed once a week and continued for 3 weeks. After euthanasia, knee joints of mice were taken for follow‐up experiments.

All mice were maintained under the following conditions: the 12 h light/12 h dark cycle, a temperature of 23 ± 1°C, and a humidity of 45%–55%. They have free access to food and water. Animal experiments were performed according to the Health Guide for the Care and Use of Laboratory Animals.

### 2.7 Quantitative real‐time polymerase chain reaction

Tissues and cells were lysed to extract total RNA using TRIpure (BioTeke). The concentration of total RNA was measured using an ultraviolet spectrophotometer (NANO 2000, Thermo Fisher). RNA was transcribed to cDNA using BeyoRT™ II M‐MLV reverse transcriptase (RNase H‐) (Beyotime) for quantitative real‐time polymerase chain reaction (qRT‐PCR). Then qRT‐PCR was performed to assess the mRNA expression levels using PCR primers (GENERAL), SYBR GREEN, and 2 × Taq PCR MasterMix (Solarbio) on Exicycler 96 system (BIONEER). For human samples, the 2^−ΔCT^ method was used to calculate the relative mRNA level. The 2^−ΔΔCT^ method was used for animal tissues or cells. Primer sequences used for qRT‐PCR were shown in Table S2.

### 2.8 Western blot analysis

The total proteins were extracted using radioimmunoprecipitation assay lysis (Solarbio) according to the manufacturer's instructions and then quantified using BCA Protein Assay Kit (Solarbio). Protein samples were separated using SDS‐PAGE and electro‐transferred to polyvinylidene fluoride membranes (Millipore). The membranes were blocked with 5% skim milk (Sangon Biotech), incubated with diluted primary antibodies at 4°C overnight, followed by diluted secondary antibodies at 37°C for 1 h. After being incubated with enhanced chemiluminescence reagent (Solarbio), the membranes were observed. The primary antibodies: AEBP1 antibody (Santa Cruz), antibodies against ADAMTS5, p‐IκBa, IκBa, p‐p65, and p65 (ABclonal), antibodies against MMP13, Aggrecan, Collagen II, and ADAMTS4 (Affinity). The secondary antibodies: Horseradish Peroxidase (HRP)‐conjugated goat anti‐rabbit or anti‐mouse IgG (Solarbio). Glyceraldehyde‐3‐phosphate dehydrogenase (GAPDH) (Proteintech) was used as an internal reference. Information on antibodies used for western blot analysis, including the dilution ratio and species origin, was listed in Table S3.

### 2.9 Co‐immunoprecipitation (Co‐IP) assay

Chondrocytes were lysed using Cell lysis buffer for Western and IP (Beyotime) to obtain the whole proteins, which were quantified using BCA Protein Assay Kit (Solarbio). Subsequently, protein samples (1 μg/μL) were incubated with AEBP1 antibody (1 μg) at 4°C overnight. Protein A agarose beads were added to conjugate antigen‐antibody complexes at 4°C for 2 h. After centrifugation, the bead‐antibody‐antigen complex was collected and washed with phosphate buffer saline. The protein‐protein complex was separated from protein A agarose beads by boiling samples. Then, the samples were centrifuged to obtain supernatant for SDS‐PAGE separation. The protein‐protein interaction was identified by western blot analysis. The whole protein sample was loaded onto SDS‐PAGE and was used as the Input control. GAPDH (Proteintech) was used as an internal reference. The original images of the blot were listed in Supporting Information S2.

### 2.10 Histological staining

The knee joints were fixed with 4% formaldehyde, dehydrated in ethanol gradient, and cleared with xylene. Then, tissues were embedded in paraffin and cut into sections (5‐μm). After being deparaffinized with xylene and rehydrated in an ethanol gradient, sections were subjected to safranin O‐Fast green staining. Safranin O‐Fast green staining was carried out through staining sections with Safranine O Cartilage Stain Solution (Solarbio) for 2 min. Finally, the stained sections were cleared with xylene, mounted with neutral gum (Sinopharm Reagent), and observed using a microscope (BX53, OLYMPUS).

### 2.11 Immunofluorescence staining

Deparaffinized and rehydrated tissue sections (5‐μm) were subjected to antigen repair at high temperature for 10 min. Cells were cultured on glass slides for immunofluorescence staining. Then, cells were fixed with 4% formaldehyde for 15 min and permeabilized with 0.1% tritonX‐100 (Beyotime) for 30 min. Then, sections or cells were blocked with 1% BSA for 15 min, and incubated with primary antibodies targeting AEBP1 (Santacruz), IκBα (Affinity), p65 (ABclonal), MMP13 (ABclonal), or Collagen II (Affinity) at 4°C overnight and secondary antibodies (Cy3‐labeled goat anti‐rabbit or anti‐mouse IgG, Invitrogen) at room temperature for 60 min. After being counterstained with DAPI (Aladdin) and treated with an anti‐fluorescence quencher (Solarbio), sections were imaged using a microscope (BX53, OLYMPUS). Information on antibodies used for immunofluorescence staining, including the dilution ratio and species origin, was listed in Table S4.

### 2.12 Ensyme‐linked immunosorbent assay

Ensyme‐linked immunosorbent assay (ELISA) was performed to determine concentrations of TNF‐α and IL‐6. The detailed procedures were performed according to the instructions of ELISA kits (LIANKE Biotech). Briefly, cells were collected and centrifuged to obtain the supernatant of culture medium. A standard, the culture medium, or the supernatant (100 μL) was added to wells of microplates. They were incubated with the antibody for TNF‐α or IL‐6 at room temperature for 2 h followed by the incubation of HRP‐labeled streptavidin at room temperature for 45 min. After color development by 3,3',5,5'‐tetramethylbenzidine, the stop solution was added for termination. The absorbance of each well was detected by a microplate reader (ELX‐800, BIOTEK).

### 2.13 Statistical analysis

All values were presented as mean ± SD. Differences between the two groups were assessed using a *t*‐test or Mann–Whitney test. One‐way ANOVA with Tukey's comparison test was performed to compare differences between three or more groups. The *p* values <0.05 were considered statistically significant.

## 3 RESULTS

### 3.1 High expression level of AEBP1 in patients with OA

**FIGURE 1:**
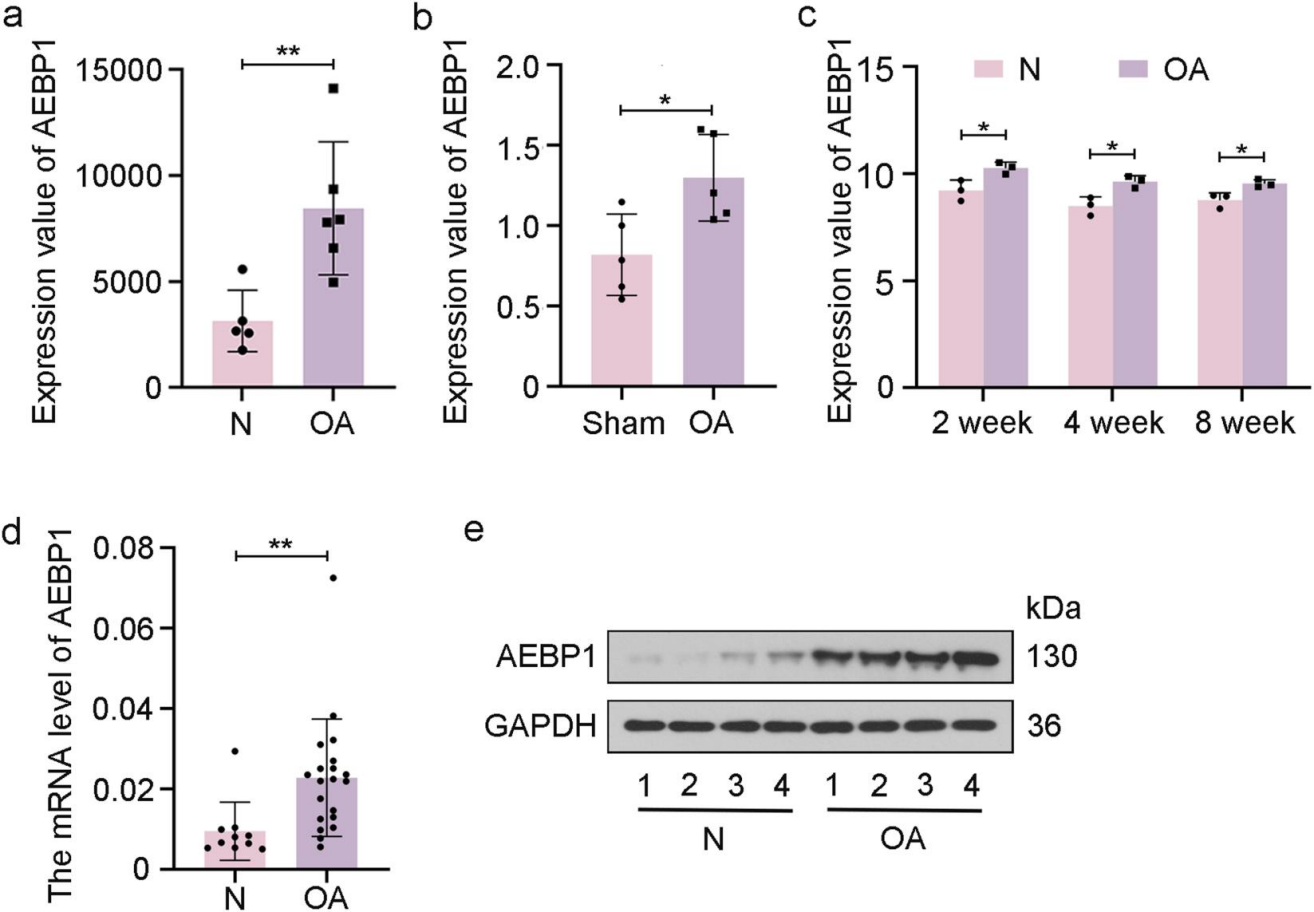
High expression level of AEBP1 in OA samples. (A) The expression value of AEBP1 in knee cartilage of normal people and patients with OA from GSE169077 dataset. (B) The expression value of AEBP1 in articular chondrocytes of sham‐ and OA‐operated rats from the GSE8077 dataset. (C) The expression value of AEBP1 in articular cartilage of untreated or OA‐operated mice at 2, 4, and 8 weeks post‐surgery from GSE53857 dataset. (D) The mRNA level of AEBP1 in articular cartilage tissues from patients with OA. (E) The protein level of AEBP1 in articular cartilage tissues from patients with OA. *p < 0.05, **p < 0.01. AEBP1, adipocyte enhancer binding protein 1; OA, osteoarthritis.

To explore the expression level of AEBP1 in OA samples, we analyzed GSE169077, GSE8077, and GSE53857 datasets. The results showed that the expression value of AEBP1 in OA samples was significantly higher than that in relatively normal samples (Figure 1A–C). The results of qRT‐PCR and western blot indicated that the mRNA and protein levels of AEBP1 were significantly increased in knee joints of patients with OA (Figure 1D,E). These results indicated that the high AEBP1 expression level might be positively associated with OA development.

### 3.2 Increased expression level of AEBP1 in mice with DMM surgery and chondrocytes with IL‐1β treatment

**FIGURE 2:**
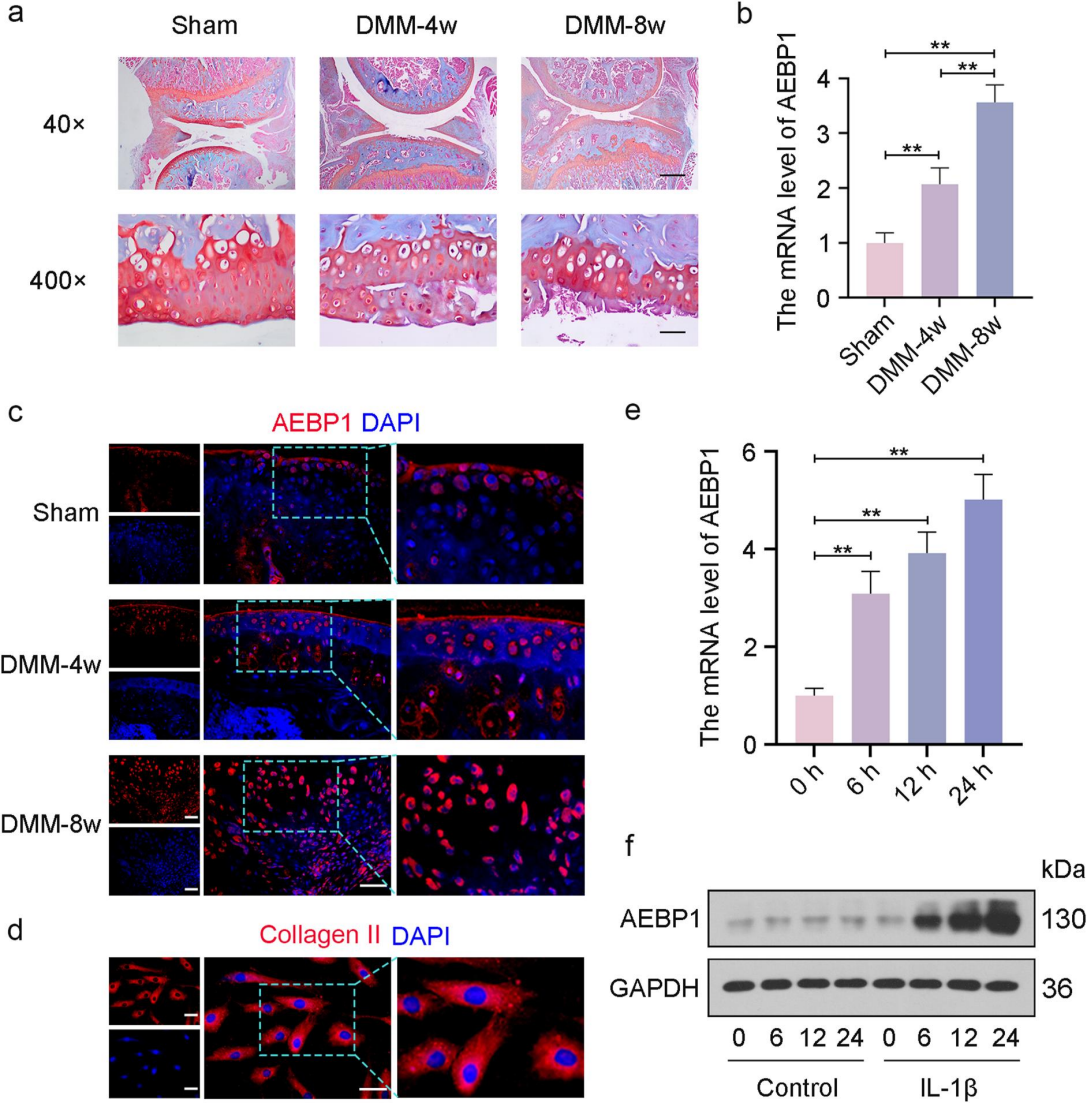
High expression level of AEBP1 in mice with OA and IL‐1β‐treated chondrocytes. (A) Representative images of safranin O‐Fast green staining of articular cartilage tissues from mice with OA. For 40×, scale bar = 500 μm; for 400×, scale bar = 50 μm. (B) The mRNA level of AEBP1 in articular cartilage tissues of mice with OA. (C) Representative images of immunofluorescence staining for AEBP1 in articular cartilage tissues of mice with OA. Scale bar = 50 μm. The focused part of the dotted frame on images was magnified to 200%. (D) Representative images of immunofluorescence staining for Collagen II in chondrocytes. Scale bar = 50 μm. The focused part of the dotted frame on images was magnified to 200%. (E) The mRNA level of AEBP1 in chondrocytes with IL‐1β treatment for 0, 6, 12, or 24 h, respectively. (F) The protein level of AEBP1 in chondrocytes with IL‐1β treatment for 0, 6, 12, or 24 h, respectively. **p < 0.01. AEBP1, adipocyte enhancer binding protein 1; OA, osteoarthritis.

To confirm the effect of AEBP1 on OA pathogenesis, the OA model was constructed in mice through DMM surgery. Safranin O fast green staining was performed to analyze the histological changes of articular cartilage. The results showed that articular cartilage of mice in the Sham group had a smooth and intact surface. Compared to mice with sham surgery, mice with DMM surgery showed a rough surface of articular cartilage and significant articular cartilage degeneration. Mice in the DMM‐8W group had more severe articular cartilage damage than mice in the DMM‐4W group (Figure 2A). The expression level of AEBP1 was significantly upregulated in mice with DMM surgery. Consistently, the AEBP1 mRNA level in the articular cartilage of mice 8 weeks after surgery is higher than those 4 weeks after surgery (Figure 2B). Further, immunofluorescence staining confirmed high AEBP1 expression level in mice with DMM surgery (Figure 2C). To mimic OA in vitro, chondrocytes were isolated from articular cartilage tissues and the Collagen II expression was identified in isolated chondrocytes. Images of immunofluorescence staining for Collagen II indicated a high expression level of Collagen II in isolated chondrocytes (Figure 2D). The in vitro OA model was induced in chondrocytes through IL‐1β treatment. Then, the expression level of AEBP1 was measured in human chondrocytes treated with IL‐1β. The mRNA and protein levels of AEBP1 were gradually elevated in the IL‐1β treatment group with treatment duration (Figure 2E,F). Consistent with the results of human samples, AEBP1 also was highly expressed in mice with OA and OA chondrocytes.

### 3.3 AEBP1 knockdown inhibited the inflammation and extracellular matrix degradation in chondrocytes with IL‐1β treatment

**FIGURE 3:**
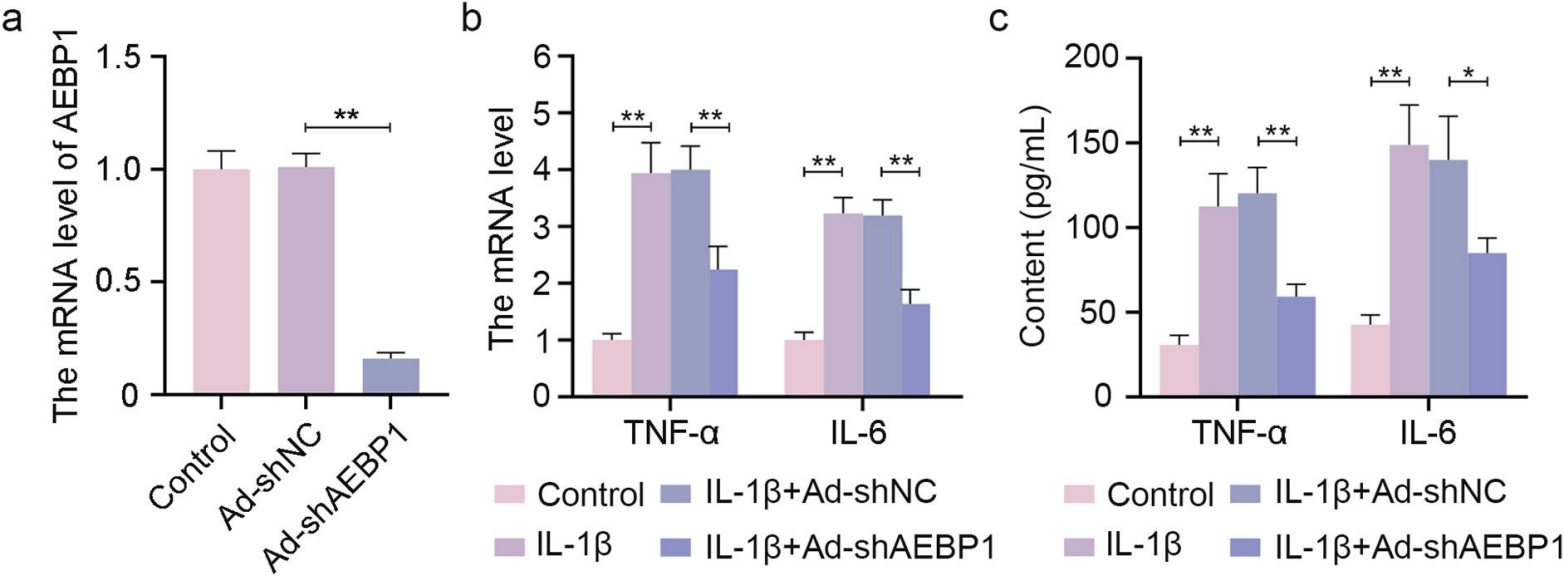
AEBP1 knockdown downregulated inflammation in IL‐1β‐treated chondrocytes. (A) The mRNA level of AEBP1 in chondrocytes after AEBP1 knockdown. (B) The mRNA levels of TNF‐α and IL‐6 in chondrocytes with IL‐1β treatment after AEBP1 knockdown. (C) The contents of TNF‐α and IL‐6 in the supernatant of chondrocytes with IL‐1β treatment after AEBP1 knockdown. *p < 0.05, **p < 0.01. AEBP1, adipocyte enhancer binding protein 1.

**FIGURE 4:**
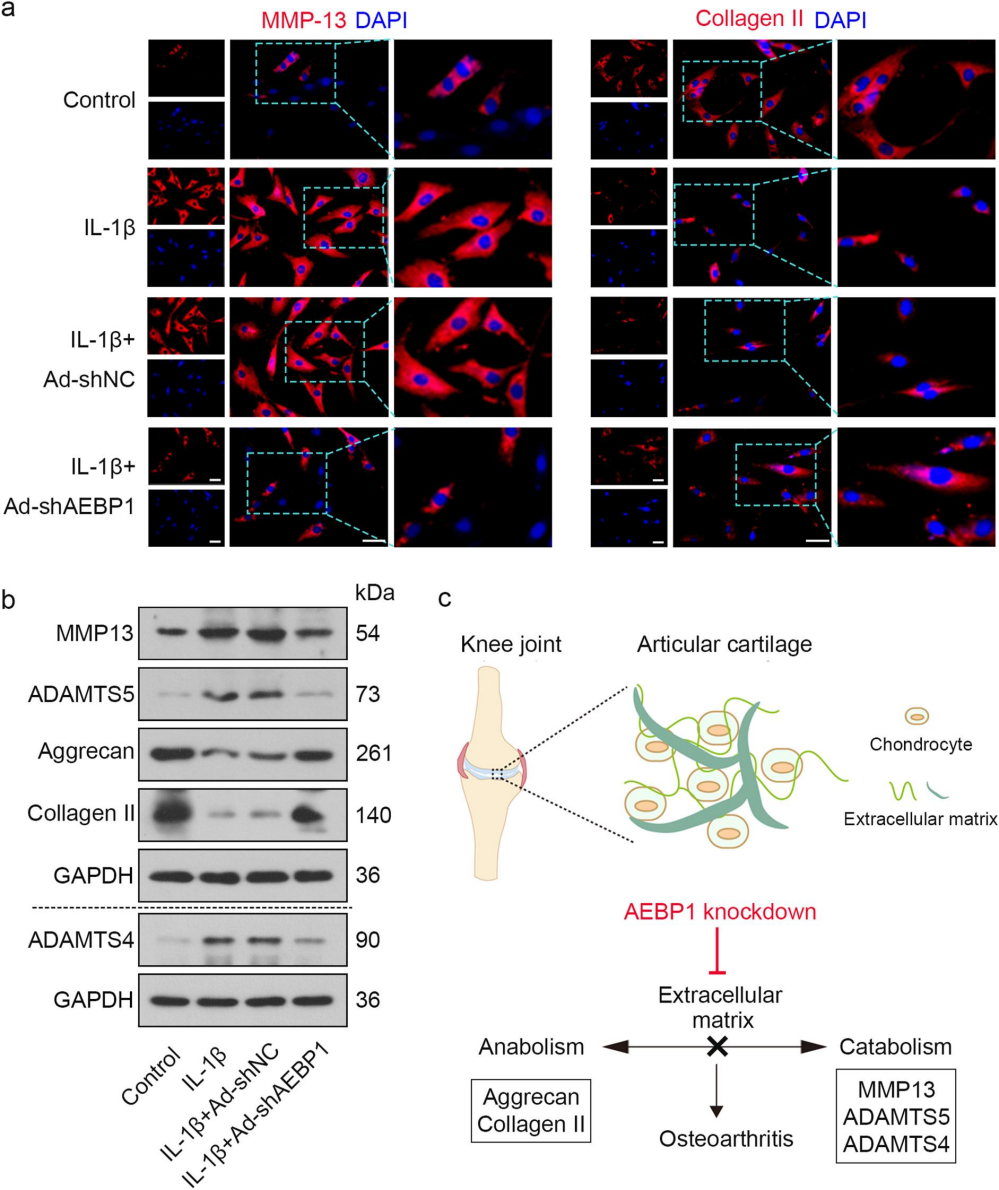
AEBP1 knockdown inhibited the degradation of extracellular matrix in IL‐1β‐treated chondrocytes. (A) Representative images of immunofluorescence staining for MMP13 and Collagen II in chondrocytes with IL‐1β treatment after AEBP1 knockdown. Scale bar = 50 μm. The focused part of the dotted frame on images was magnified to 200%. (B) The protein levels of MMP13, ADAMTS5, Aggrecan, Collagen II, and ADAMTS4 in chondrocytes with IL‐1β treatment after AEBP1 knockdown. (C) The schematic diagram of the positive effect of AEBP1 knockdown on OA through regulating the homeostasis of extracellular matrix. AEBP1, adipocyte enhancer binding protein 1; OA, osteoarthritis.

To clarify the exact role of AEBP1 in the development of OA, AEBP1 gene knockdown was performed in chondrocytes through adenovirus infection. After being infected with adenovirus, chondrocytes were collected to determine the mRNA level of AEBP1, the results showed that the expression level of AEBP1 was significantly lowered in the Ad‐shAEBP1 group (Figure 3A). Inflammation is closely related to the homeostasis of the extracellular matrix. Aggrecan and Collagen II are main components of the extracellular matrix. They can be used as anabolic markers.^20^ MMP13 is an important member of metalloproteinases, which cause the degradation of Collagen II as a class of proteinases.^21^ Of note, MMP13 at 60 kDa corresponds to its precursor form, which can be transformed into the active form (48 kDa) via an intermediate (about 55 kDa).^22^ It has been proven that the intermediate is capable of promoting the degradation of collagen protein. Therefore, MMP13 at 54 kDa was detected in the present study.^23^ ADAMTS4 and ADAMTS5 are major aggrecanases, taking part in aggrecan proteolysis.^24^ MMP13, ADAMTS4, and ADAMTS5 can be used as catabolic markers of an extracellular matrix.^25^ Infected chondrocytes were treated with IL‐1β for 24 h. After that, the mRNA levels and contents of TNF‐α and IL‐6 in chondrocytes and in the supernatant were measured by qRT‐PCR and ELISA, respectively. The results suggested that AEBP1 knockdown markedly decreased the release of TNF‐α and IL‐6 raised by IL‐1β treatment (Figure 3B,C). Images of immunofluorescence staining showed that the expression of MMP13 was considerably increased while the expression of Collagen II was decreased in the IL‐1β group, which was reversed by AEBP1 knockdown (Figure 4A). Besides, AEBP1 knockdown significantly decreased the protein levels of MMP13, ADAMTS5, and ADAMTS4, but increased the levels of Aggrecan and Collagen II (Figure 4B), indicating it regulated the homeostasis of the extracellular matrix (Figure 4C). All these results showed that AEBP1 knockdown alleviated OA through regulating inflammation and extracellular matrix degradation in chondrocytes.

### 3.4 AEBP1 knockdown increased IκBα activity and inhibited the activation of NF‐κB signaling pathway in chondrocytes with IL‐1β treatment

**FIGURE 5:**
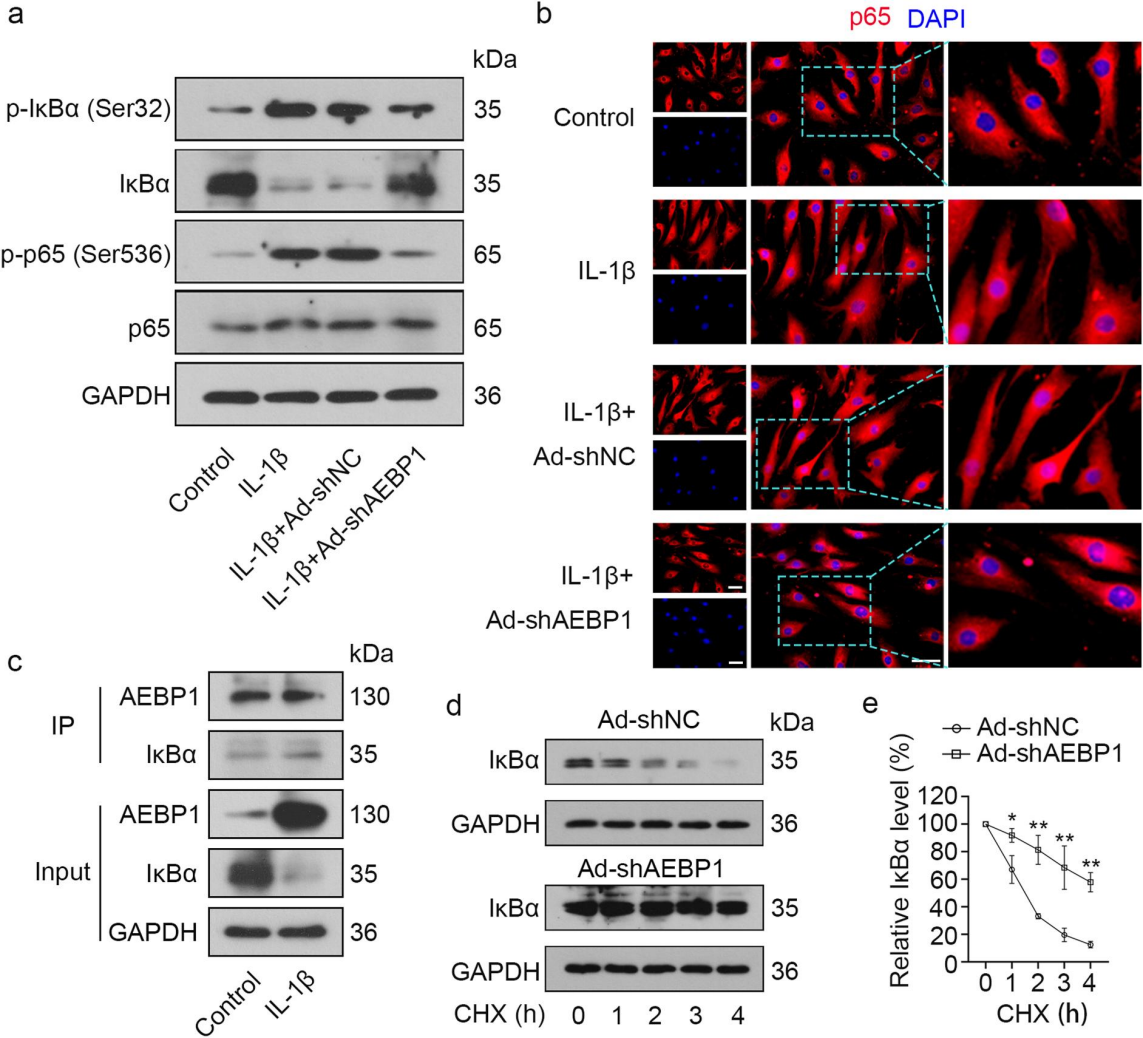
AEBP1 knockdown suppressed the activation of NF‐κB signaling pathway in IL‐1β‐treated chondrocytes. (A) The protein levels of p‐IκBα (Ser32), IκBα, p‐p65 (Ser536), and p65 in chondrocytes with IL‐1β treatment after AEBP1 knockdown. (B) Representative images of immunofluorescence staining for p65 in chondrocytes with IL‐1β treatment after AEBP1 knockdown. Scale bar = 50 μm. The focused part of the dotted frame on images was magnified to 200%. (C) The interaction between AEBP1 and IκBα was assessed by a Co‐IP assay. (D) The protein level of IκBα in chondrocytes treated with CHX for 0, 1, 2, 3, or 4 h. (E) The protein level of IκBα in chondrocytes treated with CHX after AEBP1 knockdown. *p < 0.05, **p < 0.01. AEBP1, adipocyte enhancer binding protein 1; CHX, cycloheximide; Co‐IP, co‐immunoprecipitation; OA, osteoarthritis.

NF‐κB signaling pathway is closely related to OA progression through regulating the expression levels of MMP13 and Collagen II.^26^ Thus, we investigated whether the NF‐κB signaling pathway was activated in IL‐1β‐treated chondrocytes after AEBP1 knockdown. The results showed that IL‐1β treatment significantly elevated the protein levels of p‐IκBα (Ser32) and p‐p65 (Ser536), but markedly reduced the protein levels of IκBα in chondrocytes, all these changes were reversed by AEBP1 knockdown (Figure 5A). Immunofluorescence staining was used to assess the nuclear translocation of p65 and the images showed that obvious nuclear translocation of p65 in chondrocytes treated with IL‐1β, while AEBP1 knockdown suppressed the nuclear translocation of p65 (Figure 5B), suggesting that AEBP1 knockdown inhibited the activation of NF‐κB signaling pathway. AEBP1 has been reported to bind to IκBα,^15^ which could inhibit the activation of NF‐κB.^27^ Thus, we further investigated whether the role of AEBP1 knockdown in inhibiting the activation of NF‐κB pathway was associated with IκBα. Through performing a Co‐IP assay in chondrocytes, we found that AEBP1 interacted with IκBα to the same extent under the basal and IL‐1β conditions. The input control also confirmed that higher AEBP1 expression induced by IL‐1β negatively regulated the IκBα expression (Figure 5C). Then infected chondrocytes were treated with CHX for 0, 1, 2, 3, or 4 h, respectively. The protein level of IκBα was measured using western blot to determine the effect of AEBP1 knockdown on the relative level of IκBα. AEBP1 knockdown contributed to the maintenance of IκBα protein stability (Figure 5D,E). Taken together, these results suggested that AEBP1 knockdown inhibited the activation of NF‐κB pathway through increasing the protein activity of IκBα in chondrocytes treated with IL‐1β.

### 3.5 IκBα knockdown depleted the effect of AEBP1 knockdown on inflammation and extracellular matrix degradation in IL‐1β‐treated chondrocytes

**FIGURE 6:**
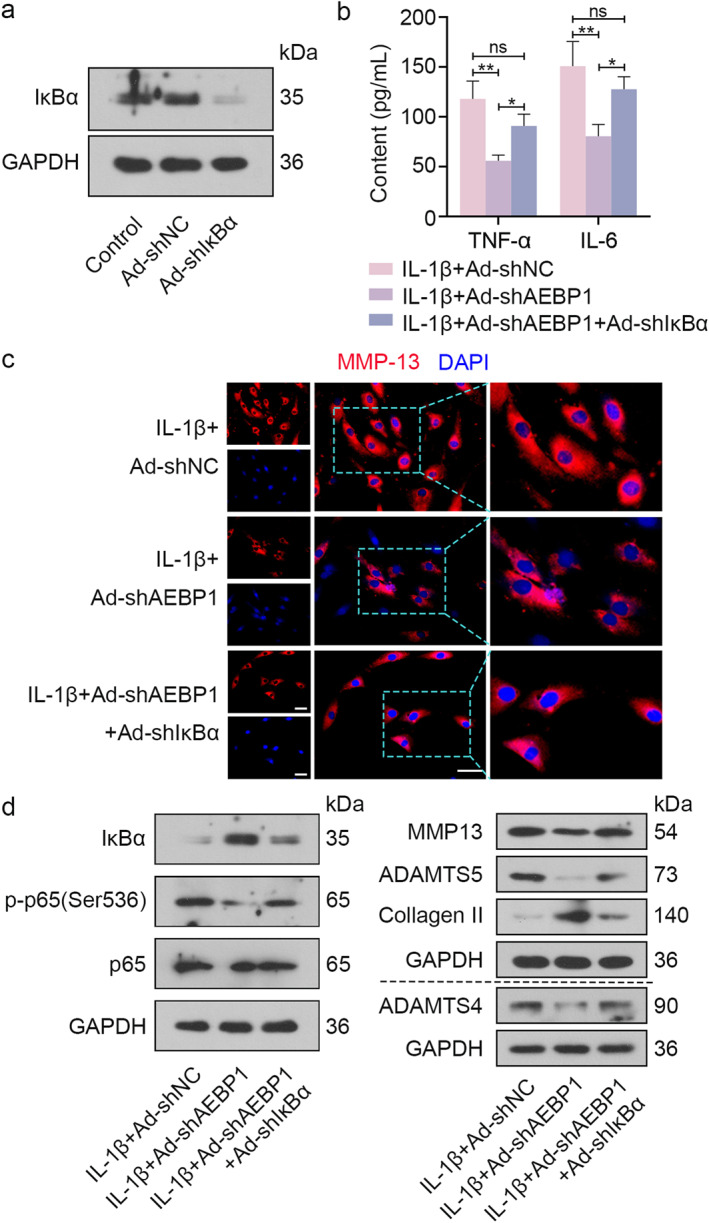
IκBα knockdown removed the protective role of AEBP1 knockdown on IL‐1β‐treated chondrocytes. (A) The protein level of IκBα in chondrocytes after IκBα knockdown. (B) The contents of TNF‐α and IL‐6 in the supernatant of chondrocytes with IL‐1β treatment were determined by ELISA after AEBP1 and IκBα knockdown. (C) Representative images of immunofluorescence staining for MMP13 in chondrocytes with IL‐1β treatment after AEBP1 and IκBα knockdown. Scale bar = 50 μm. The focused part of the dotted frame on images was magnified to 200%. (D) The protein levels of IκBα, p‐p65 (Ser536), p65, MMP13, ADAMTS5, Collagen II, and ADAMTS4 in chondrocytes with IL‐1β treatment after AEBP1 and IκBα knockdown. *p < 0.05, **p < 0.01. AEBP1, adipocyte enhancer binding protein 1; ELISA, ensyme‐linked immunosorbent assay; ns means no significance.

Then, IκBα knockdown in chondrocytes was performed to further test the crucial role of IκBα. The results of western blot indicated the protein level of IκBα was significantly decreased after IκBα knockdown (Figure 6A). Adenovirus carrying shIκBα and shAEBP1 were used to co‐infect chondrocytes. After infection, chondrocytes were stimulated with IL‐1β for 24 h. Subsequently, contents of TNF‐α and IL‐6 in the culture supernatant were measured by ELISA. We found that IκBα knockdown depleted the effect of AEBP1 knockdown on decreasing the levels of TNF‐α and IL‐6 in IL‐1β‐stimulated chondrocytes (Figure 6B). Images of immunofluorescence staining of MMP13 suggested that AEBP1 and IκBα knockdown increased the expression of MMP13 (Figure 6C). Western blot analysis showed the decreased expression levels of IκBα and Collagen II, while the increased expression levels of p‐p65 (Ser536), MMP13, ADAMTS5, and ADAMTS4 in the IL‐1β + Ad‐shAEBP1 + Ad‐shIκBα group compared with the IL‐1β + Ad‐shAEBP1 group (Figure 6D). All these results indicated that the role of AEBP1 knockdown in inhibiting activation of NF‐κB pathway was dependent on the increase of IκBα.

### 3.6 AEBP1 knockdown alleviated OA in mice through increasing IκBα activity and inhibiting activation of NF‐κB signaling pathway

**FIGURE 7:**
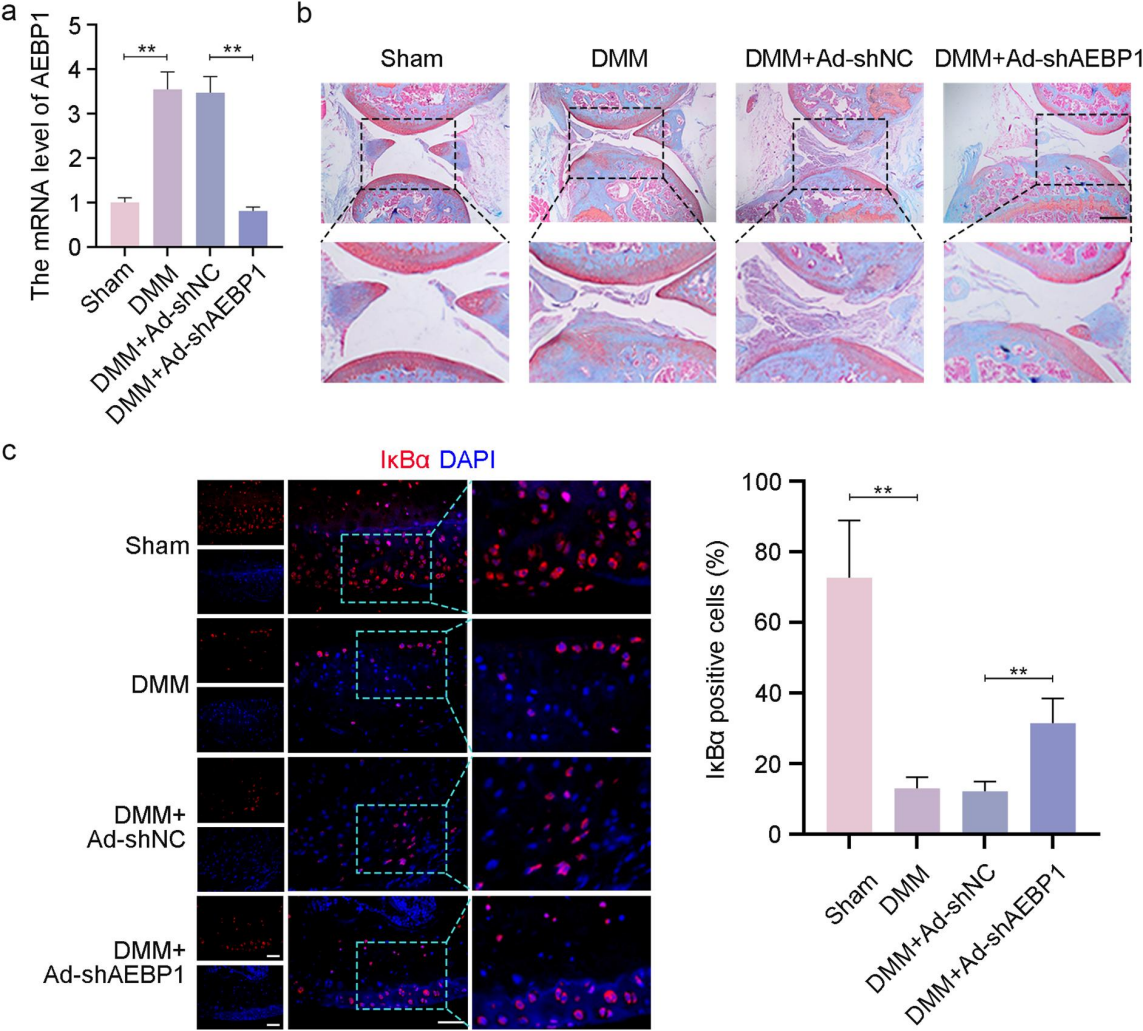
Intra‐articular injection of chondrocytes with AEBP1 knockdown alleviated OA in mice. (A) The mRNA level of AEBP1 in articular cartilage tissues from mice with OA. (B) Representative images of safranin O‐Fast green staining of articular cartilage tissues from mice with OA. Scale bar = 500 μm. The focused part of the dotted frame on images was magnified to 200%. (C) Representative images and quantitative analysis of immunofluorescence staining for IκBα in articular cartilage tissues from mice with OA. Scale bar = 50 μm. The focused part of the dotted frame on images was magnified to 200%. **p < 0.01. AEBP1, adipocyte enhancer binding protein 1; OA, osteoarthritis.

The effect of AEBP1 knockdown on the development of OA was also investigated in mice with OA through intra‐articular injection of adenovirus carrying shAEBP1. The expression level of AEBP1 in articular cartilage was significantly increased in mice with DMM surgery. However, AEBP1 knockdown significantly decreased the expression level of AEBP1 (Figure 7A). The images of safranin O‐Fast green staining showed obvious articular cartilage degeneration in mice with OA. Conversely, AEBP1 knockdown partially restored articular cartilage degradation caused by DMM surgery (Figure 7B). Next, we performed immunofluorescence staining of articular cartilage tissues to localize IκBα expression. Quantitative analysis suggested that the expression of IκBα was significantly decreased in the DMM group. AEBP1 knockdown markedly elevated the expression of IκBα (Figure 7C). These results confirmed that AEBP1 knockdown exerted a protective role in mice with OA by increasing IκBα expression.

## 4 DISCUSSION

In this study, we explored the impact of AEBP1 on the development of OA. Our results showed a high expression level of AEBP1 in the articular cartilage of OA individuals, IL‐1β‐stimulated chondrocytes, and mice with DMM surgery. AEBP1 knockdown improved OA by increasing the protein activity of IκBα, inhibiting the activation of NF‐κB pathway, down regulating inflammation, and suppressing extracellular matrix degradation in IL‐1β‐stimulated chondrocytes. The in vitro improvement of AEBP1 knockdown was depleted after IκBα knockdown. The protective role of AEBP1 knockdown was also confirmed in mice with OA.

OA has posed a heavy burden to numerous patients due to pain and disability. Although great progress has been made in recent years, diagnosis for OA is relatively difficult. It is often completed by evaluating the degree of injury, such as the joint space, or bone defect. Severer injury corresponds to a higher degree of OA.^28^ The current treatments for OA are mainly surgical techniques, which usually present age‐related outcomes. For microfracture and osteochondral autograft transfer, younger patients have better prognosis than older patients. Emerging treatment technologies for OA partly concentrate on the field of tissue engineering, including synthesis of intracellular scaffolds from synthetic or natural materials, expansion of chondrocytes in vitro, and culture of meniscal fibro‐chondrocytes. All these advanced treatments could provide long‐term strategies for OA mitigation. However, redifferentiation of dedifferentiated cells in vivo seems to be a problem.^7^ OA involves degenerative changes in the entire joint.^29^ Articular cartilage could primarily affect joint function and its damage is one of the typical characteristics of OA.^30^, ^31^ Therefore, regulating the homeostatic state of articular cartilage remains ongoing improvement in research for OA treatment. Wang et al.^30^ reported that FoxO1 overexpression protected against OA through maintaining the homeostasis of articular cartilage. In our research, we also tried to delay the development of OA based on the thinking of maintaining the homeostasis of articular cartilage, which relies on the chondrocytes and the extracellular matrix.

Inflammation has been proven to be an important risk factor in OA progression.^32^ It leads to aberrant chondrocyte metabolism which drives the changes in extracellular matrix.^33^ AEBP1, a transcriptional repressor, closely relates to multiple inflammatory diseases. AEBP1 promoted the development of colon adenocarcinoma through activating NF‐κB pathway.^34^ The expression level of AEBP1 was also upregulated in non‐alcoholic steatohepatitis fibrosis characterized by hepatic inflammation.^35^ Through database analysis, we found that the expression level of AEBP1 was significantly elevated in rats with OA. Then human subjects were recruited for further assessment. Consistently, the expression level of AEBP1 in articular cartilage of OA patients was higher than that of the control. Furthermore, aging also is one of the risk factors for OA.^3^ Through comparing the expression level of AEBP1 in mice or chondrocytes, we also found the expression level of AEBP1 was positively correlated with the duration and severity of OA. Our results substantially confirmed that AEBP1 significantly increased in OA samples. Although obesity is a risk factor of OA, the AEBP1 mRNA tended to be upregulated in female offspring of rats fed a 40% fat diet compared with those fed a 10% fat diet,^36^ AEBP1 was able to regulate adipocyte differentiation,^37^ the OA models studied in this study do not involve obesity or abnormal lipid metabolism, thus this study did not explore whether the effect of AEBP1 on OA was related to lipid metabolism.^37^

Common methods inducing OA in vivo mostly include DMM surgery, anterior cruciate ligament transection (ACLT) surgery, and sodium iodoacetate injection. Nowadays, DMM surgery is widely used to induce OA in mice due to its high reliability, excellent reproducibility, and characteristics structurally similar to human OA.^38^ ACLT also is a familiar method to induce OA. Compared with DMM surgery, ACLT surgery is a milder means of inducing OA. Obvious OA‐like changes can only be seen after a longer cycle in ACLT models.^39^ The OA model induced by injection of sodium iodoacetate showed more obvious pain characteristics.^40^ IL‐1β treatment is almost a universal method to induce OA in chondrocytes. Its experimental principle is related to the degradation of the extracellular matrix.^41^ Based on the structural similarity between DMM surgery‐induced OA and human OA, and the general use of IL‐1β treatment, DMM surgery‐induced OA in mice and IL‐1β‐induced OA in chondrocytes were constructed for determining the expression level of AEBP1 in OA. Increased AEBP1 expression also was observed in mice with OA and IL‐1β‐treated chondrocytes. Of note, only male mice used for the animal experiments was a limitation of the current study. Hwang et al.^42^ reported that both male and female mice undergoing DMM surgery showed obvious cartilage degeneration. Furthermore, the present study aimed to explore the underlying mechanism by which AEBP1 affects articular cartilage homeostasis in OA progression. Therefore, we did not pay more attention to the impact of sex difference on OA. Sex differences need to be carefully considered in our future experiments involving OA.

In view of the substance of inflammation in OA progression,^32^ chondrocytes of AEBP1 knockdown were constructed through adenovirus infection for evaluating the changes of inflammation and extracellular matrix. We found that AEBP1 knockdown contributed to the decreased protein levels of p‐IκBα (Ser32) and p‐p65 (Ser536), the increased protein levels of IκBα. Besides, the reduced nuclear translocation of p65 was observed in the AEBP1 knockdown group. All these results indicated that AEBP1 knockdown suppressed the activation of NF‐κB pathway. Several studies suggested that the activation of NF‐κB signaling pathway promoted by AEBP1 was implicated in the decreased expression level of IκBα. Guo et al.^43^ reported that AEBP1 decreased IκBα expression by upregulating its phosphorylation, activating NF‐κB pathway. It was well‐established that the IκB kinase (IKK) complex recognizes and phosphorylates IκBα, thereby promoting its ubiquitination and degradation.^44^ A prospective study demonstrated that AEBP1 had no influence on IKK activity and is not a part of the IKK complex. AEBP1 physically interacted with IκBα by its discoidin‐like domain, which made IκBα easier to be phosphorylated and subsequently degraded.^45^ In addition, Ren et al.^15^ suggested that AEBP1 was bound to IκBα and further activated NF‐κB signaling pathway. In the current study, we also verified the interaction between AEBP1 and IκBα. AEBP1 knockdown could sustain IκBα protein stability. Therefore, we supposed that the inhibitory role of AEBP1 knockdown on NF‐κB might be attributed to that it protected IκBα against phosphorylation and degradation, suppressing the nuclear translocation of NF‐κB.

In conclusion, our findings demonstrated that the increased AEBP1 expression level was correlated with OA progression. AEBP1 promoted the development of OA through decreasing IκBα activity, activating NF‐κB signaling pathway, upregulating inflammation, and contributing to the degradation of the extracellular matrix.

## 5 CONCLUSIONS

In the present study, we explored the association between AEBP1 and OA through analyzing articular cartilage of patients with OA or mice with DMM surgery and IL‐1β‐treated chondrocytes. We found that the high AEBP1 expression was positively related to the development of OA. AEBP1 knockdown downregulated inflammation and degradation of the extracellular matrix in IL‐1β‐treated chondrocytes. AEBP1 knockdown protects against OA, which might be mediated by the increased IκBα activity and the inhibition of NF‐κB signaling pathway. Further, IκBα knockdown depleted the positive effect of AEBP1 knockdown on IL‐1β‐treated chondrocytes. AEBP1 knockdown alleviated OA progression also was confirmed in mice with DMM surgery. Collectively, our findings showed that AEBP1 promotes the development of OA through activating NF‐κB signaling pathway‐mediated inflammation and degradation of extracellular matrix.

## AUTHOR CONTRIBUTIONS

All authors contributed to the study conception and design. Le Cao and Weilu Gao performed the experiments, data collection, and wrote the first draft of the manuscript. Haitao Yang and Ran Zeng performed data analysis and figure preparation. Zongsheng Yin revised the manuscript and supervised the study.

## CONFLICT OF INTEREST STATEMENT

The authors declare that there is no conflict of interest regarding the publication of this article.

## ETHICS STATEMENT

All experimental procedures were approved by Anhui Medical University. All patients participating in our study have signed informed consent and all experiment protocols were performed according to the Declaration of Helsinki. Animal experiments were performed according to the Health Guide for the Care and Use of Laboratory Animals.

## Supporting information

Supporting Information S1

Supporting Information S2

## Data Availability

The data that support the findings of this study are available from the corresponding author upon reasonable request.
