# Supplementary material for: Adipocyte enhancer binding protein 1 knockdown alleviates osteoarthritis through inhibiting NF‐κB signaling pathway‐mediated inflammation and extracellular matrix degradation
Source: J Cell Commun Signal. 2024 Mar 22;18(2):e12022. doi: 10.1002/ccs3.12022 (PMC11208125; doi:10.1002/ccs3.12022)
Supplement: Supplementary file 1 — Supporting Information S1 [file CCS3-18-e12022-s002.docx]

Journal of Cell Communication and Signaling

# AEBP1 knockdown alleviates osteoarthritis through inhibiting NF-κB signaling pathway-mediated inflammation and extracellular matrix degradation

Le Cao^a, b^; Weilu Gao^a^; Haitao Yang^b^; Ran Zeng^c, *^; Zongsheng Yin^a, *^

*Correspondence:

Zongsheng Yin, Email: [yinzs1961@163.com](mailto:yinzs1961@163.com); Department of Orthopedics, the First Affiliated Hospital of Anhui Medical University, No. 218, Jixi Road, Hefei, Anhui, China.

Ran Zeng, Email: [caoranran147@163.com](mailto:caoranran147@163.com); Department of Intensive Care Unit, Fuyang Hospital of Anhui Medical University, No. 99, Huangshan Road, Fuyang, Anhui, China.

***Supplementary information***

**Table S1 Information of human samples.**

| Gene | Normal (N) | Osteoarthritis (OA) |
| --- | --- | --- |
| Number of patients | 10 | 20 |
| Number of female | 3 | 15 |
| Number of male | 7 | 5 |
| Age distribution (year) | 56~83 | 52~81 |
| Mean age (year) | 69.70 ± 7.945 | 67.40 ± 9.445 |

**Table S2 Primer sequences used for qRT-PCR.**

| Gene | Forward sequence | Reverse sequence |
| --- | --- | --- |
| mus AEBP1 | CTCCCTGAGCCAGTTGTG | TGTATGTGCGAGTGATTGTG |
| homo AEBP1 | AGACCACGCCATCTTCCG | CCTTGTTGTTCTCCCACTCG |
| homo TNF-α | GAGTGACAAGCCTGTAGCC | AAGAGGACCTGGGAGTAGAT |
| homo IL-6 | GTCCAGTTGCCTTCTCCC | GCCTCTTTGCTGCTTTCA |
| mus GAPDH | TGTTCCTACCCCCAATGTGTCCGTC | CTGGTCCTCAGTGTAGCCCAAGATG |
| homo GAPDH | GACCTGACCTGCCGTCTAG | AGGAGTGGGTGTCGCTGT |

**Table S3 Antibodies used for western blot.**

| Primary antibodies (Abs) | Species origin of Abs | Secondary Abs |
| --- | --- | --- |
| AEBP1 Ab (1:500) | Mouse | Goat-anti-mouse IgG-HRP (1:3000) |
| MMP13 Ab (1:1000) | Rabbit | Goat-anti-rabbit IgG-HRP (1:3000) |
| ADAMTS5 Ab (1:500) |  |  |
| Aggrecan Ab (1:400) |  |  |
| Collagen-II Ab (1:500) |  |  |
| p-IκBα Ab (1:1000) |  |  |
| IκBα Ab (1:1000) |  |  |
| p-p65 Ab (1:1000) |  |  |
| p65 Ab (1:500) |  |  |
| ADAMTS4 Ab (1:500) |  |  |
| GAPDH Ab (1:10000) | Mouse | Goat-anti-mouse IgG-HRP (1:3000) |

**Table S4 Antibodies used for immunofluorescence staining.**

| Primary antibodies (Abs) | Species origin of Abs | Secondary Abs |
| --- | --- | --- |
| AEBP1 Ab (1:50) | Mouse | Goat-anti-mouse IgG-Cy3 (1:200) |
| IκBα Ab (1:50) | Rabbit | Goat-anti-rabbit IgG-Cy3 (1:200) |
| p65 Ab (1:100) |  |  |
| MMP13 Ab (1:100) |  |  |
| Collagen II Ab (1:100) |  |  |
