# Supplementary material for: Adipocyte enhancer binding protein 1 knockdown alleviates osteoarthritis through inhibiting NF‐κB signaling pathway‐mediated inflammation and extracellular matrix degradation
Source: J Cell Commun Signal. 2024 Mar 22;18(2):e12022. doi: 10.1002/ccs3.12022 (PMC11208125; doi:10.1002/ccs3.12022)
Supplement: Supplementary file 2 — Supporting Information S2 [file CCS3-18-e12022-s001.pptx]

## Slide 1
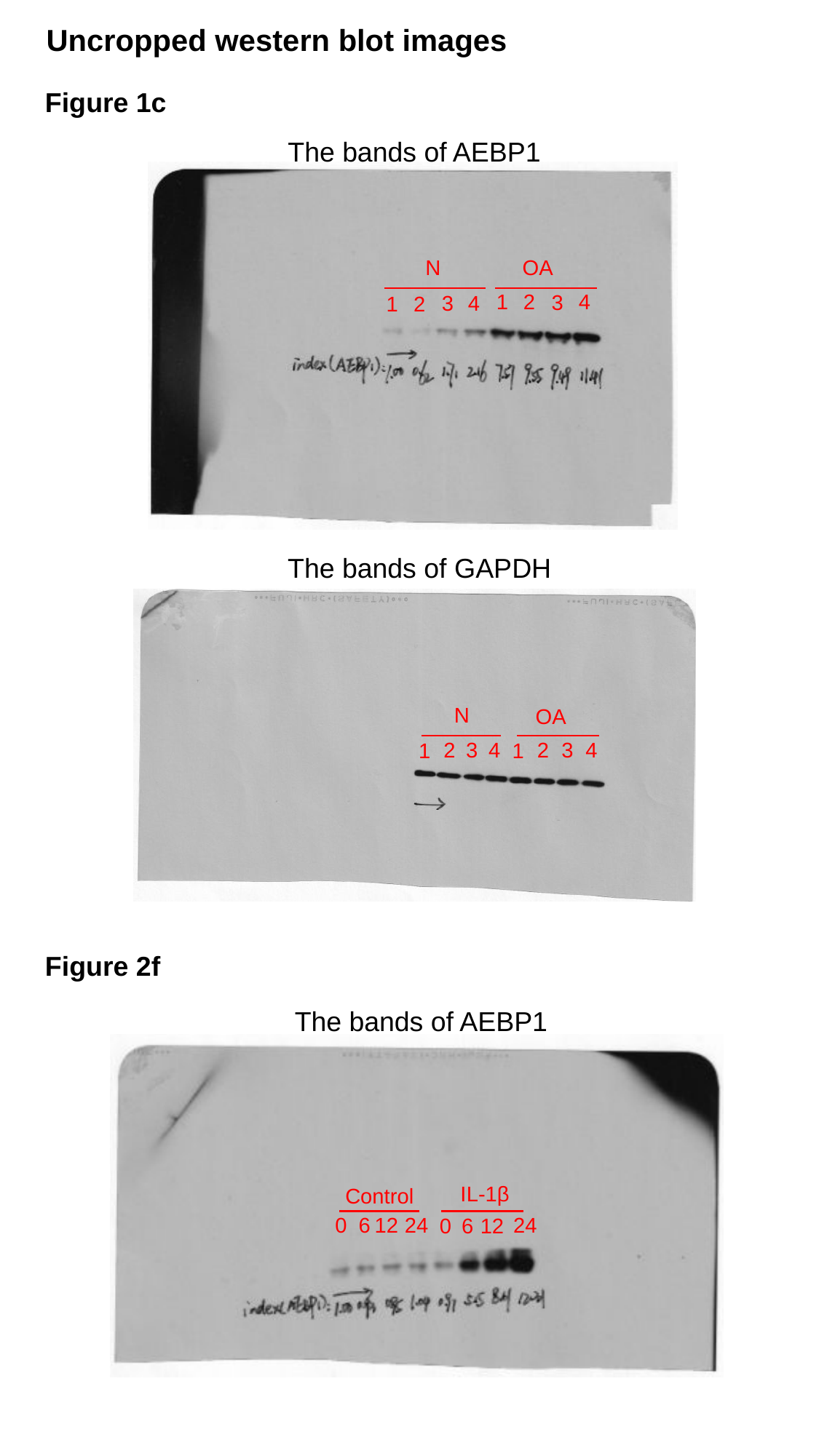

Uncropped western blot images
Figure 1c
The bands of AEBP1
N
OA
4
2
1
3
3
4
2
1
The bands of GAPDH
N
OA
2
3
4
4
3
2
1
1
Figure 2f
The bands of AEBP1
IL-1β
Control
6
24
12
0
24
0
6
12

## Slide 2
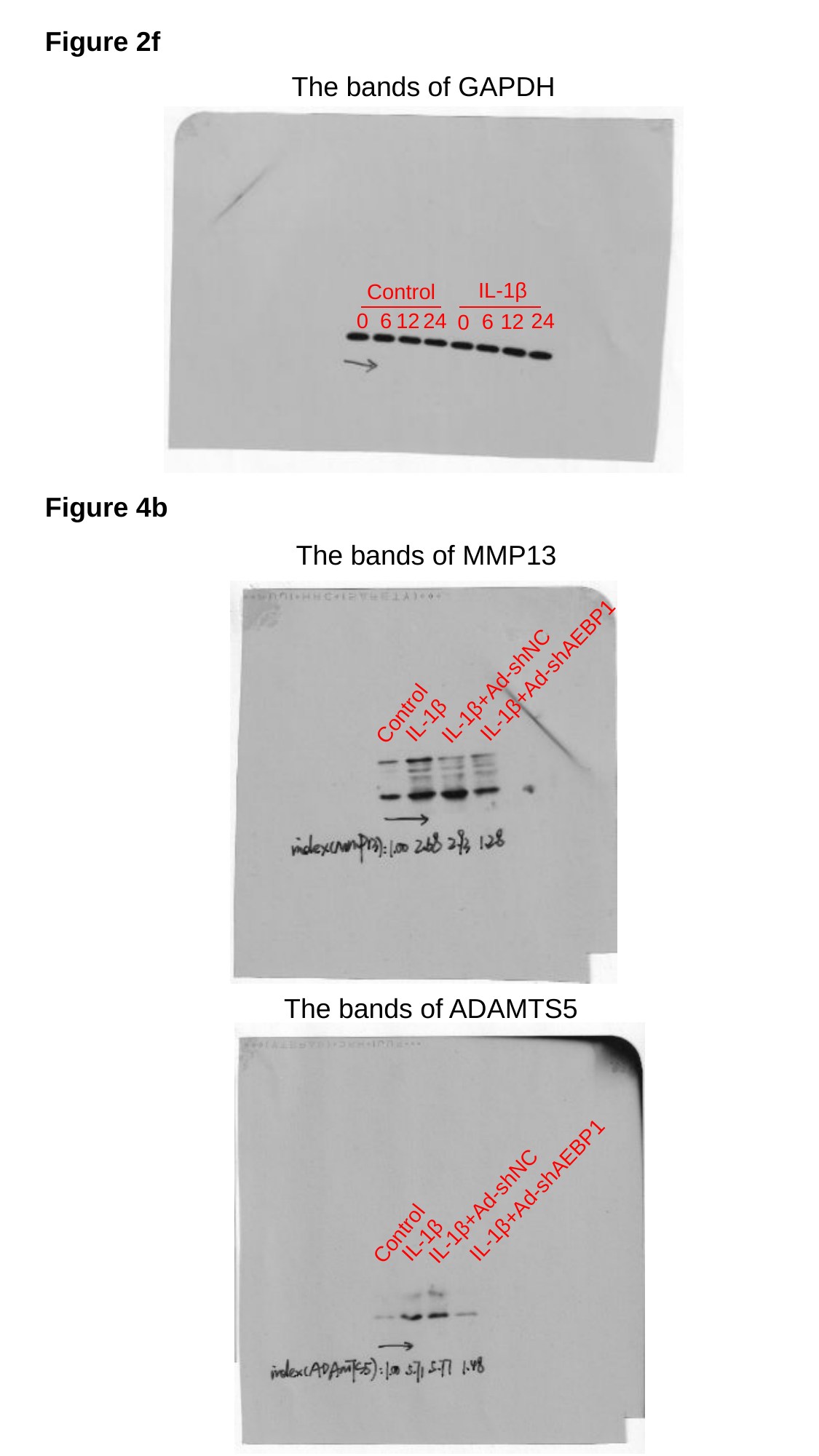

Figure 2f
The bands of GAPDH
IL-1β
Control
24
6
24
12
0
6
12
0
Figure 4b
The bands of MMP13
IL-1β+Ad-shAEBP1
IL-1β+Ad-shNC
Control
IL-1β
The bands of ADAMTS5
IL-1β+Ad-shAEBP1
IL-1β+Ad-shNC
Control
IL-1β

## Slide 3
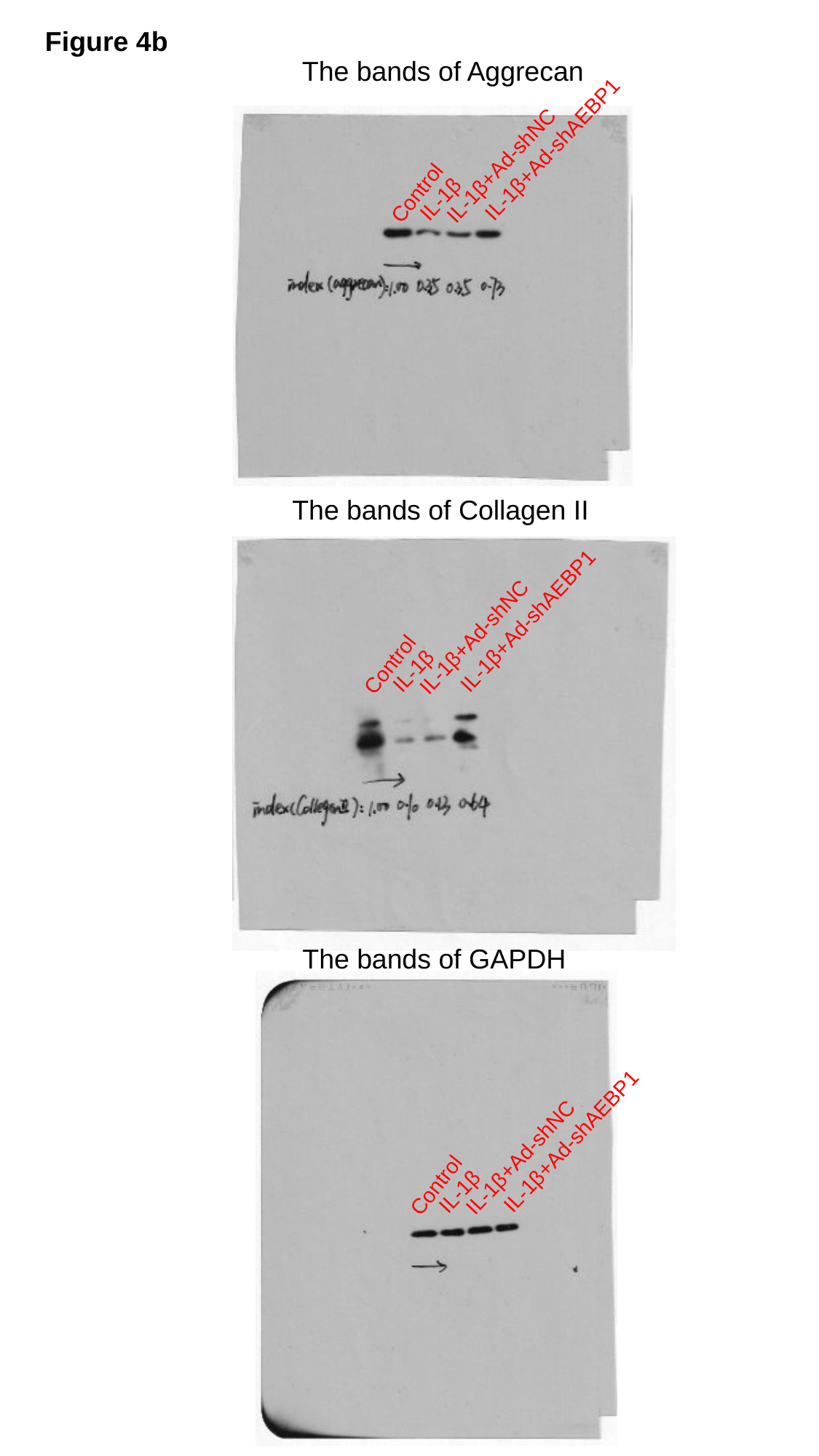

Figure 4b
The bands of Aggrecan
IL-1β+Ad-shAEBP1
IL-1β+Ad-shNC
Control
IL-1β
The bands of Collagen II
IL-1β+Ad-shAEBP1
IL-1β+Ad-shNC
Control
IL-1β
The bands of GAPDH
IL-1β+Ad-shAEBP1
IL-1β+Ad-shNC
Control
IL-1β

## Slide 4
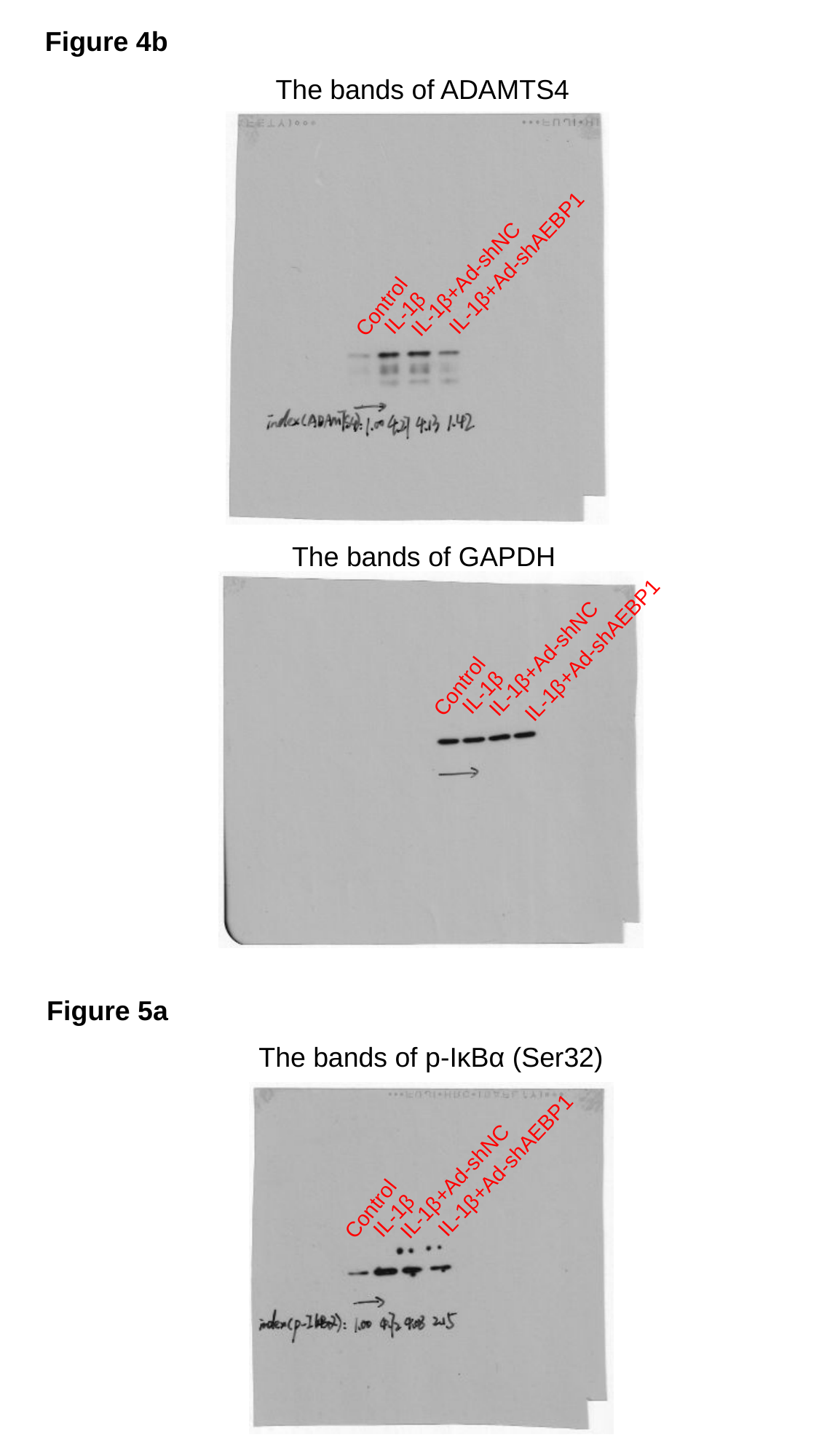

Figure 4b
The bands of ADAMTS4
IL-1β+Ad-shAEBP1
IL-1β+Ad-shNC
Control
IL-1β
The bands of GAPDH
IL-1β+Ad-shAEBP1
IL-1β+Ad-shNC
Control
IL-1β
Figure 5a
The bands of p-IκBα (Ser32)
IL-1β+Ad-shAEBP1
IL-1β+Ad-shNC
Control
IL-1β

## Slide 5
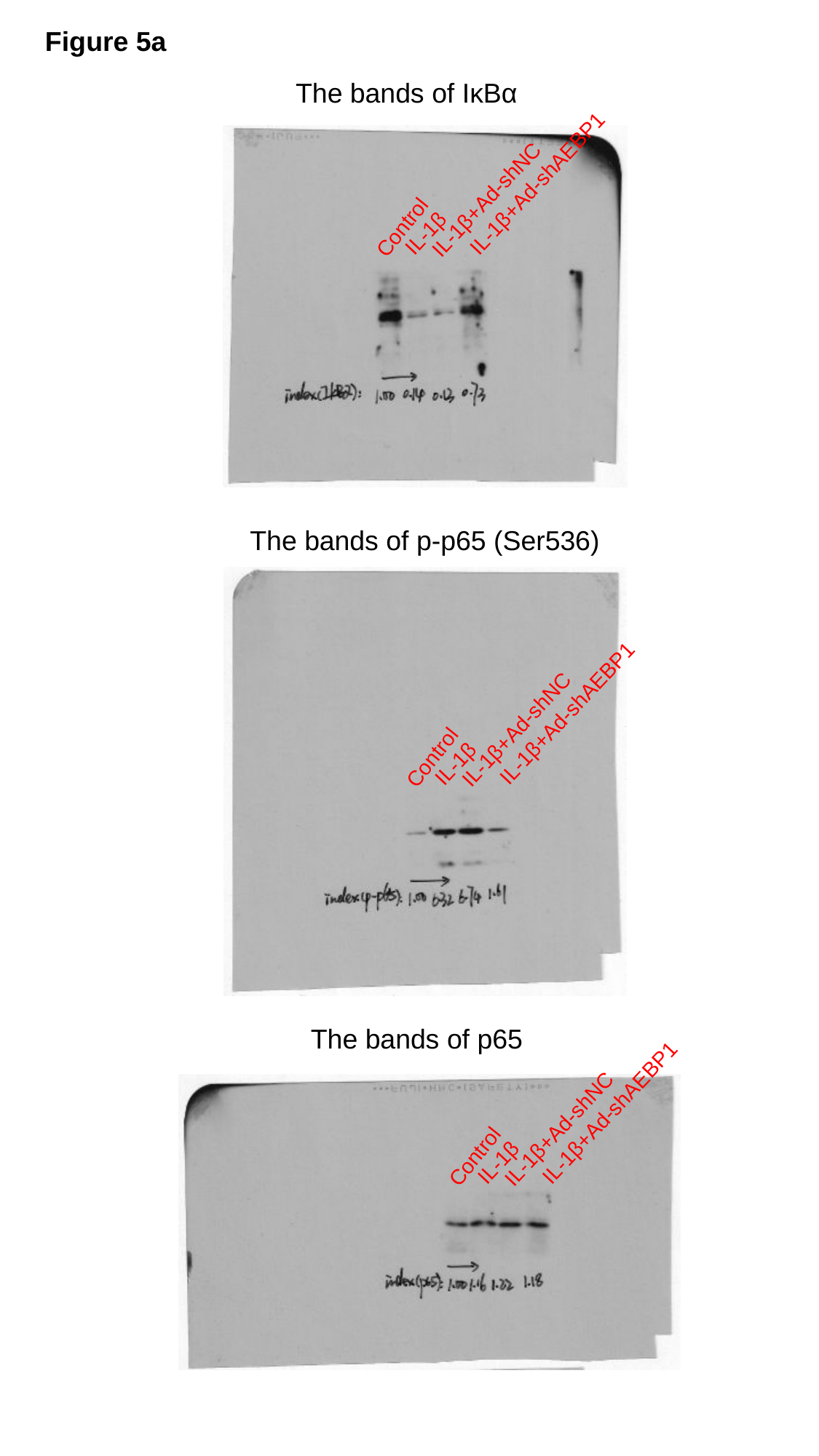

Figure 5a
The bands of IκBα
IL-1β+Ad-shAEBP1
IL-1β+Ad-shNC
Control
IL-1β
The bands of p-p65 (Ser536)
IL-1β+Ad-shAEBP1
IL-1β+Ad-shNC
Control
IL-1β
The bands of p65
IL-1β+Ad-shAEBP1
IL-1β+Ad-shNC
Control
IL-1β

## Slide 6
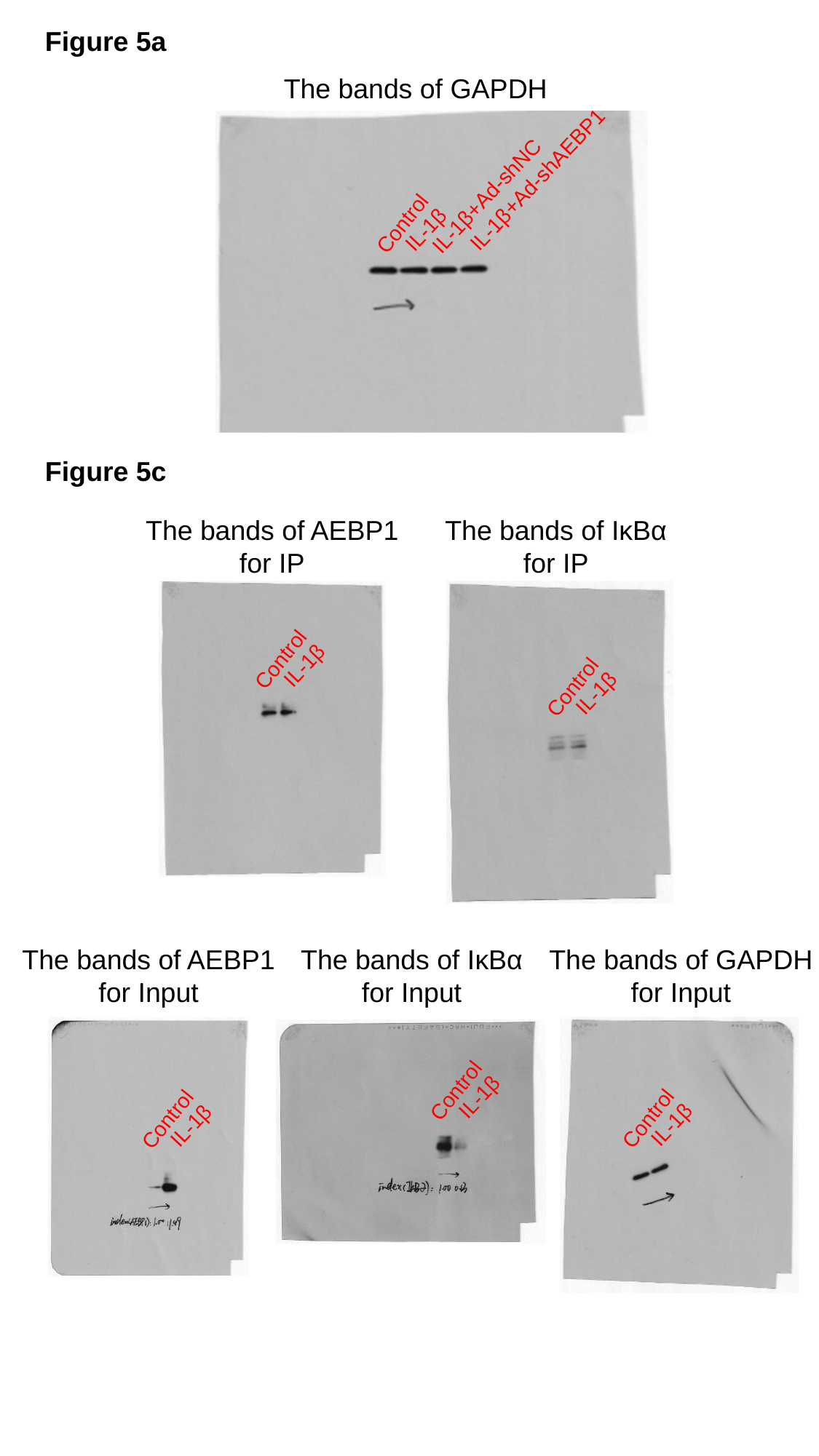

Figure 5a
The bands of GAPDH
IL-1β+Ad-shAEBP1
IL-1β+Ad-shNC
Control
IL-1β
Figure 5c
The bands of AEBP1
for IP
The bands of IκBα
for IP
Control
IL-1β
Control
IL-1β
The bands of GAPDH
for Input
The bands of IκBα
for Input
The bands of AEBP1
for Input
Control
IL-1β
Control
Control
IL-1β
IL-1β

## Slide 7
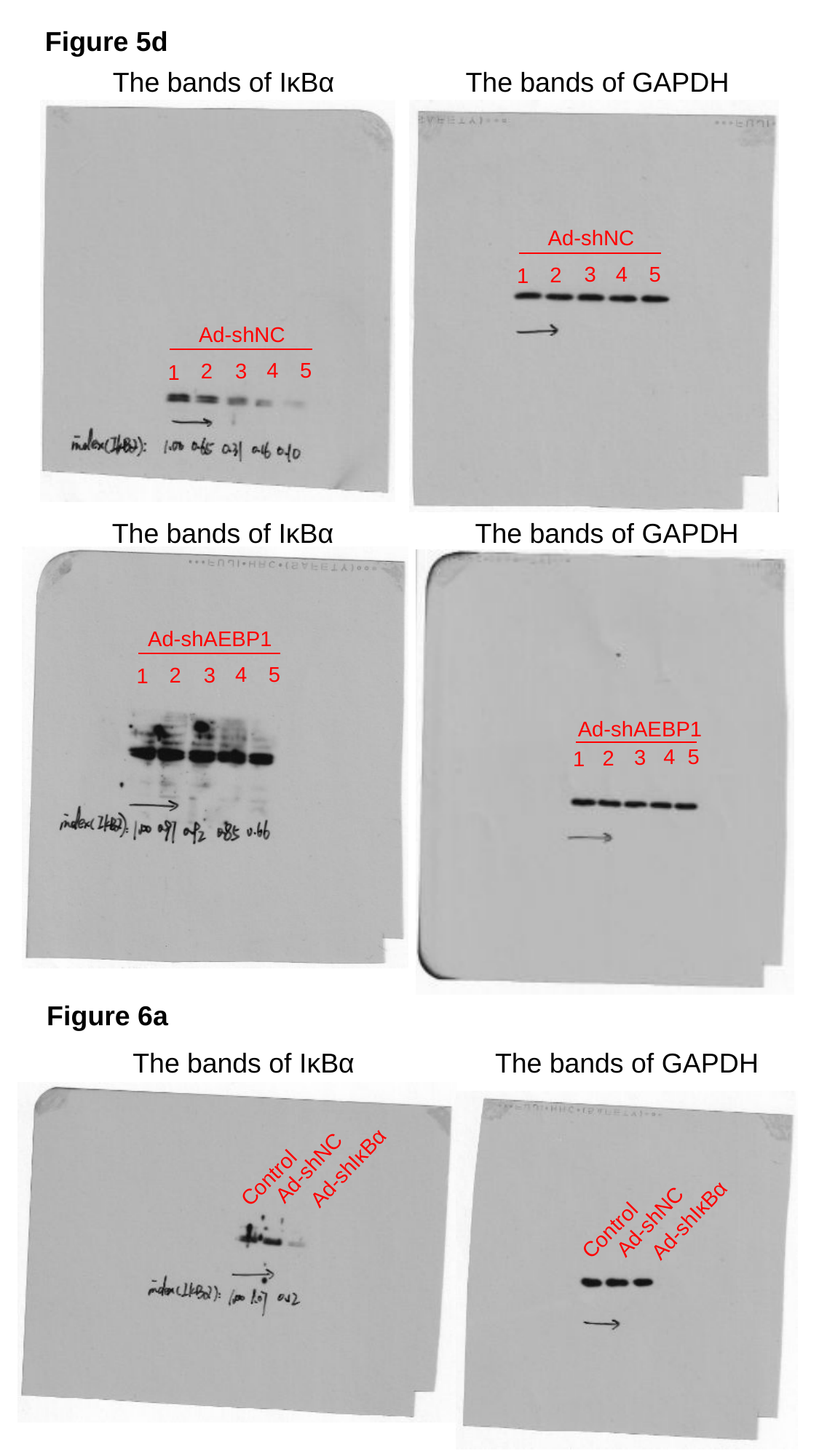

Figure 5d
The bands of IκBα
The bands of GAPDH
Ad-shNC
4
5
3
2
1
Ad-shNC
4
5
3
2
1
The bands of IκBα
The bands of GAPDH
Ad-shAEBP1
4
5
3
2
1
Ad-shAEBP1
4
5
3
2
1
Figure 6a
The bands of IκBα
The bands of GAPDH
Ad-shNC
Ad-shIκBα
Control
Ad-shIκBα
Ad-shNC
Control

## Slide 8
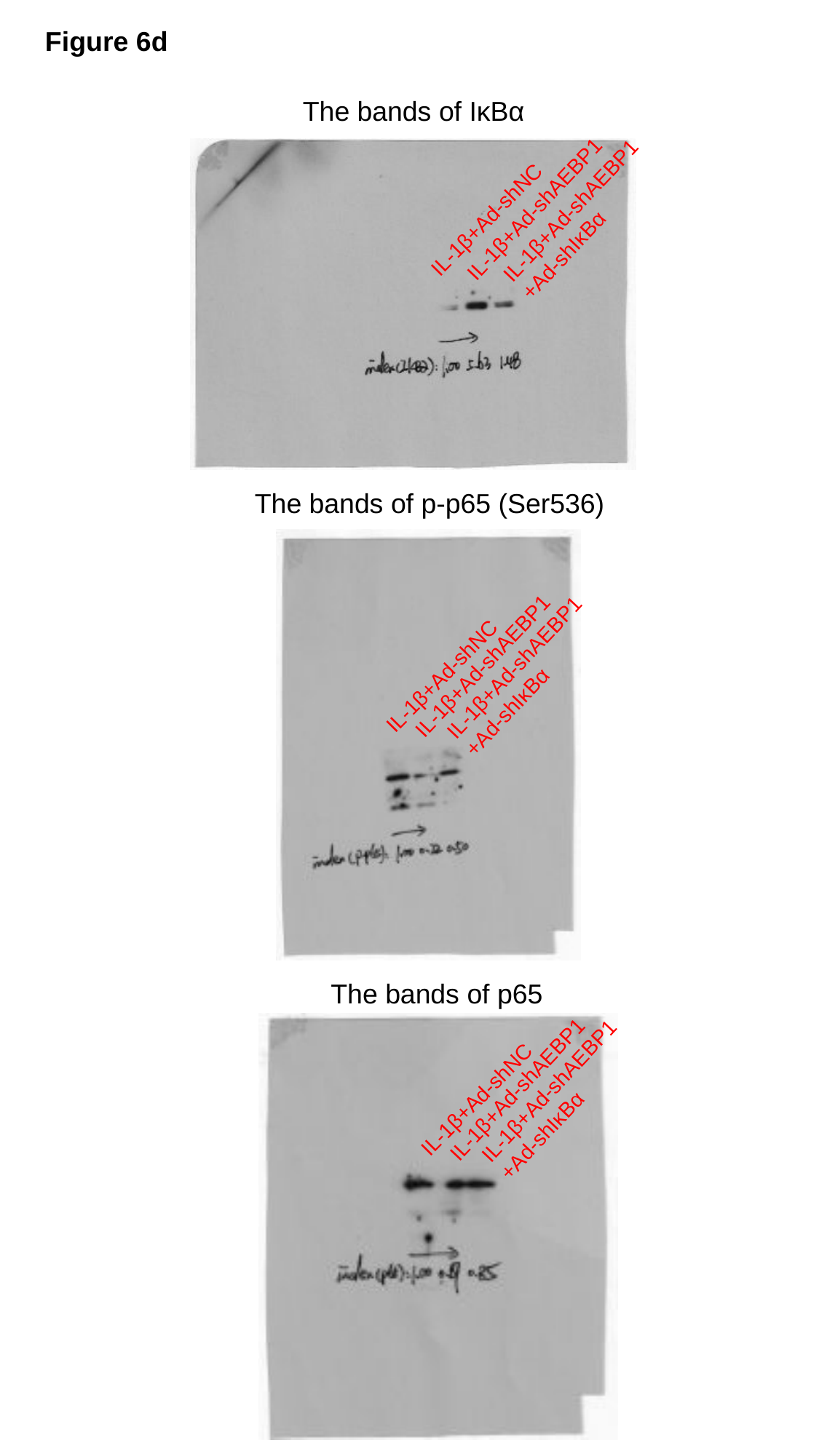

Figure 6d
The bands of IκBα
IL-1β+Ad-shAEBP1
+Ad-shIκBα
IL-1β+Ad-shAEBP1
IL-1β+Ad-shNC
The bands of p-p65 (Ser536)
IL-1β+Ad-shAEBP1
+Ad-shIκBα
IL-1β+Ad-shAEBP1
IL-1β+Ad-shNC
The bands of p65
IL-1β+Ad-shAEBP1
+Ad-shIκBα
IL-1β+Ad-shAEBP1
IL-1β+Ad-shNC

## Slide 9
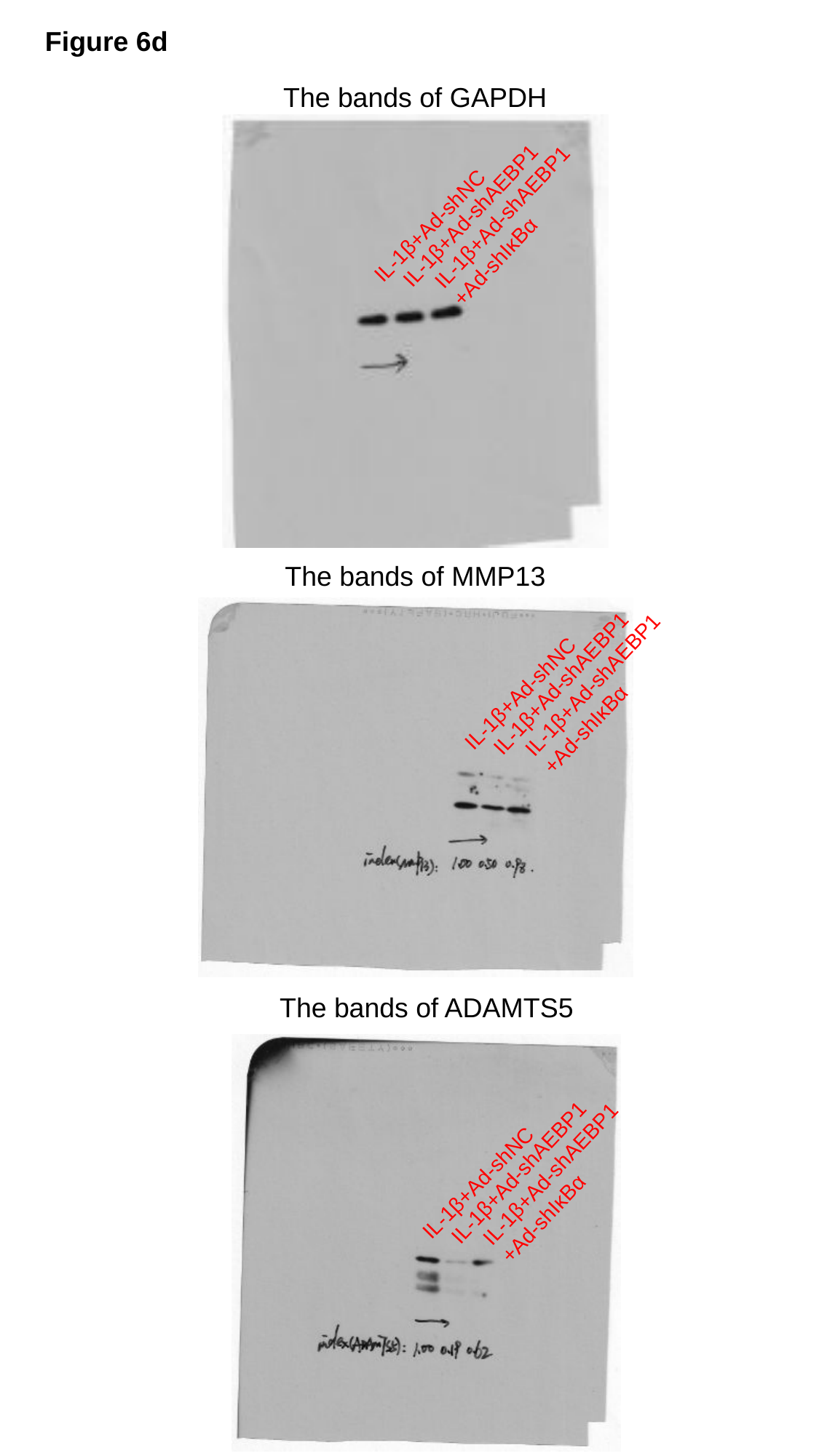

Figure 6d
The bands of GAPDH
IL-1β+Ad-shAEBP1
+Ad-shIκBα
IL-1β+Ad-shAEBP1
IL-1β+Ad-shNC
The bands of MMP13
IL-1β+Ad-shAEBP1
+Ad-shIκBα
IL-1β+Ad-shAEBP1
IL-1β+Ad-shNC
The bands of ADAMTS5
IL-1β+Ad-shAEBP1
+Ad-shIκBα
IL-1β+Ad-shAEBP1
IL-1β+Ad-shNC

## Slide 10
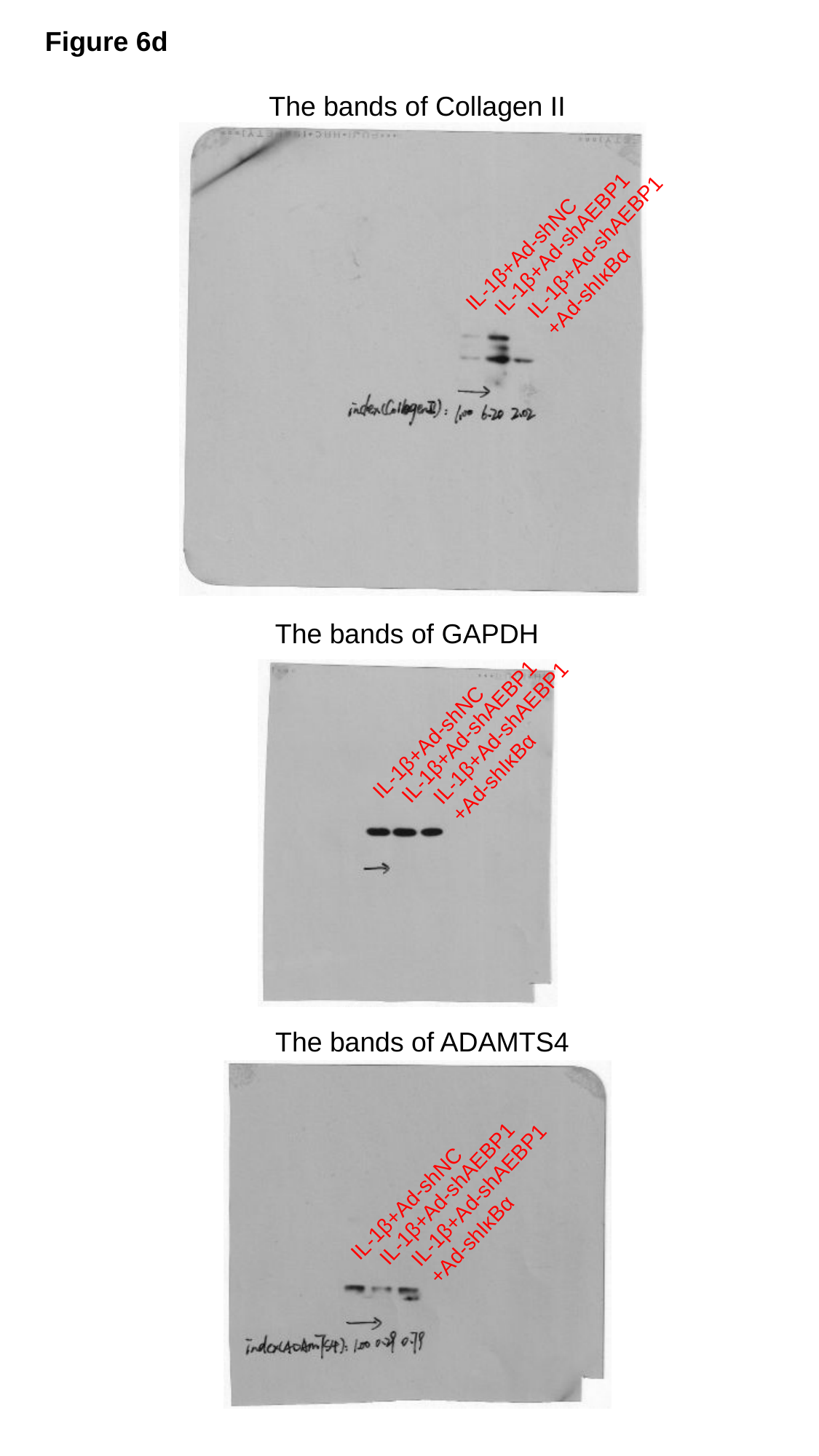

Figure 6d
The bands of Collagen II
IL-1β+Ad-shAEBP1
+Ad-shIκBα
IL-1β+Ad-shAEBP1
IL-1β+Ad-shNC
The bands of GAPDH
IL-1β+Ad-shAEBP1
+Ad-shIκBα
IL-1β+Ad-shAEBP1
IL-1β+Ad-shNC
The bands of ADAMTS4
IL-1β+Ad-shAEBP1
+Ad-shIκBα
IL-1β+Ad-shAEBP1
IL-1β+Ad-shNC

## Slide 11
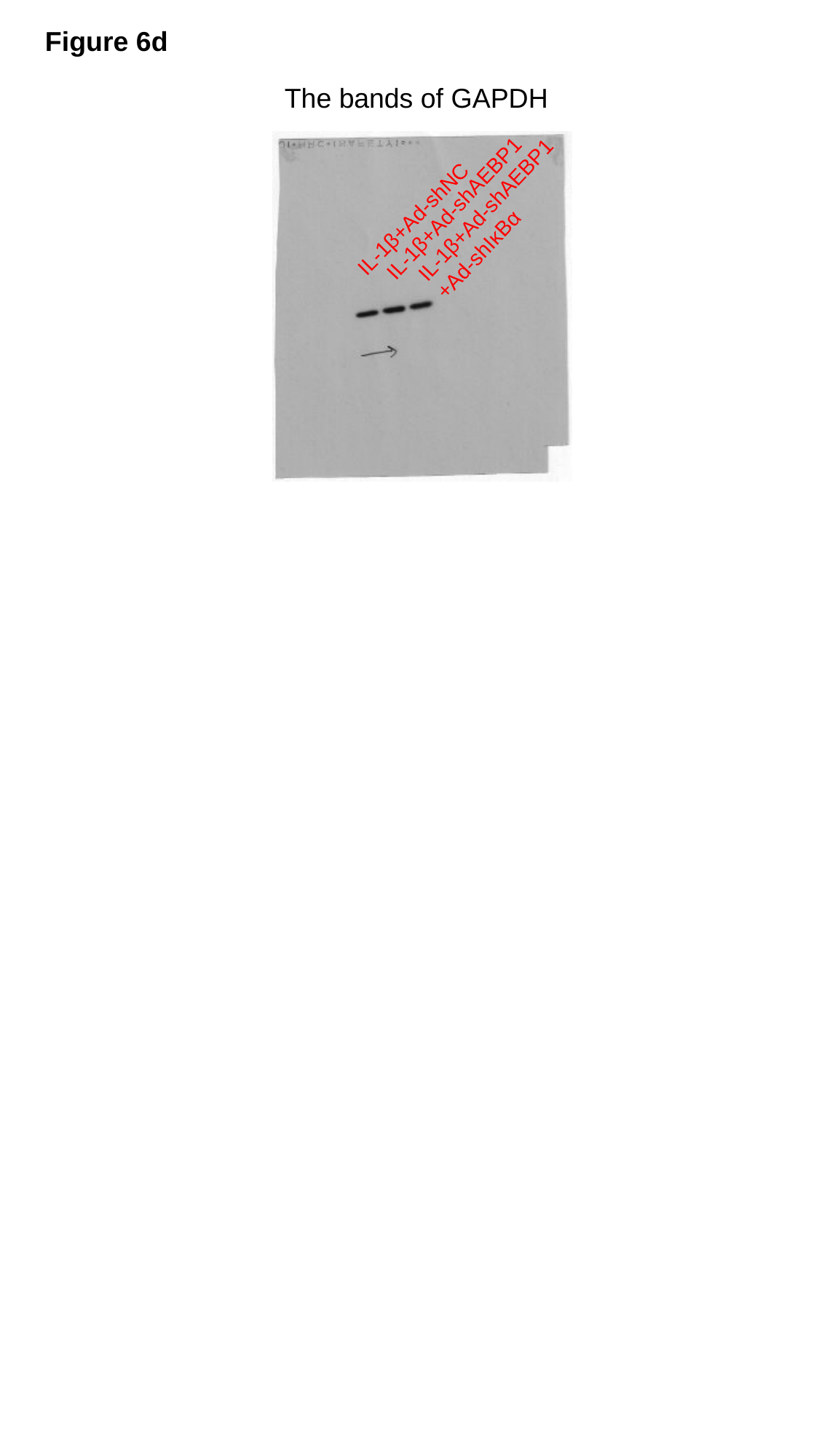

Figure 6d
The bands of GAPDH
IL-1β+Ad-shAEBP1
+Ad-shIκBα
IL-1β+Ad-shAEBP1
IL-1β+Ad-shNC
